# Home rehabilitation supported by a wearable soft-robotic device for improving hand function in older adults: A pilot randomized controlled trial

**DOI:** 10.1371/journal.pone.0220544

**Published:** 2019-08-06

**Authors:** Bob Radder, Gerdienke B. Prange-Lasonder, Anke I. R. Kottink, Johnny Holmberg, Kristin Sletta, Manon van Dijk, Thomas Meyer, Alejandro Melendez-Calderon, Jaap H. Buurke, Johan S. Rietman

**Affiliations:** 1 Roessingh Research and Development, Enschede, the Netherlands; 2 Department of Biomechanical Engineering, University of Twente, Enschede, the Netherlands; 3 Department of Biosystems and Signals, University of Twente, Enschede, the Netherlands; 4 Eskilstuna Kommun Vård- och omsorgsförvaltningen, Eskilstuna, Sweden; 5 National Foundation for the Elderly, Bunnik, the Netherlands; 6 terzStiftung, Berlingen, Switzerland; 7 Department of Physical Medicine and Rehabilitation, Northwestern University, Chicago, IL, United States of America; 8 Cereneo Advanced Rehabilitation Institute, Vitznau, Switzerland; Kurume University School of Medicine, JAPAN

## Abstract

**Background:**

New developments, based on the concept of wearable soft-robotic devices, make it possible to support impaired hand function during the performance of daily activities and intensive task-specific training. The wearable soft-robotic ironHand glove is such a system that supports grip strength during the performance of daily activities and hand training exercises at home.

**Design:**

This pilot randomized controlled clinical study explored the effect of prolonged use of the assistive ironHand glove during daily activities at home, in comparison to its use as a trainings tool at home, on functional performance of the hand.

**Methods:**

In total, 91 older adults with self-perceived decline of hand function participated in this study. They were randomly assigned to a 4-weeks intervention of either assistive or therapeutic ironHand use, or control group (received no additional exercise or treatment). All participants performed a maximal pinch grip test, Box and Blocks test (BBT), Jebsen-Taylor Hand Function Test (JTHFT) at baseline and after 4-weeks of intervention. Only participants of the assistive and therapeutic group completed the System Usability Scale (SUS) after the intervention period.

**Results:**

Participants of the assistive and therapeutic group reported high scores on the SUS (mean = 73, SEM = 2). The therapeutic group showed improvements in unsupported handgrip strength (mean Δ = 3) and pinch strength (mean Δ = 0.5) after 4 weeks of ironHand use (p≤0.039). Scores on the BBT and JTHFT improved not only after 4 weeks of ironHand use (assistive and therapeutic), but also in the control group. Only handgrip strength improved more in the therapeutic group compared to the assistive and control group. No significant correlations were found between changes in performance and assistive or therapeutic ironHand use (p≥0.062).

**Conclusion:**

This study showed that support of the wearable soft-robotic ironHand system either as assistive device or as training tool may be a promising way to counter functional hand function decline associated with ageing.

## Introduction

Hand function predominantly determines the quality of performance in activities of daily living (ADL) and work-related functioning. Older adults with age-related loss of muscle mass (i.e. sarcopenia) [1] and/or age-related diseases (e.g. stroke, arthritis) [2, 3] suffer from loss of hand function. As a consequence, they experience functional limitations, which affects independence in performing ADL [3–5].

An effective intervention for improving hand function of (stroke) patients should consist of several key aspects of motor learning, such as high-intensity and task-specificity in repetitive and functional exercises that are actively initiated by the patient him/herself [6, 7]. In a traditional rehabilitation setting, those kinds of interventions are performed with one-on-one attention from the healthcare professional for each patient. This might become problematic in the near future when the population of older adults with age-related diseases (e.g. stroke, rheumatoid arthritis) with hand function decline will rise, resulting in an increased need for healthcare professionals and a rise of healthcare costs [8]. Therefore, new alternatives to provide intensive therapy for all patients are needed in the future.

New technological developments, such as robot-assisted hand training, have the potential to provide such intensive, repetitive and task-specific therapy. Several reviews [9–11] already showed positive results on motor function after robot-assisted training of the upper extremity. However, limiting factors of robot-assisted therapy are the need for supervision of a healthcare professional, the high costs of the devices and the limited availability of wearable devices for training at home [12]. Furthermore, it is often not efficient in transferring the trained movements into daily situations [6]. Therefore, the next generation robotic training approaches should pay substantial attention towards home-based rehabilitation and the functional nature of the exercise involved.

A new way of providing functional, intensive and task-specific hand training would involve using new technological innovations that enable support of the affected hand directly during the performance of ADL, based on the concept of a wearable robotic glove [13–18]. In this way, the affected hand can be used repeatedly and for prolonged periods of time during functional daily activities. These robotic gloves can use different human-robot interfaces to provide assistance for the affected hand, such as an EMG-controlled glove, a tendon driven glove, a glove controlled by force sensors etc. [13, 14, 16, 18, 19]. All these robotic gloves use soft and flexible materials to make such devices more lightweight and easy to use, accommodating wearable applications. This concept of a wearable soft-robotic glove allows persons with reduced hand function to use their hand(s) during a large variety of functional activities and may even turn performing daily activities into extensive training, independent from the availability of healthcare professionals. This is thought to improve hand function and patient’s independence in performing ADL.

Therefore, an easy to use and wearable soft-robotic glove (ironHand system), supporting grip strength and hand training exercises at home, was developed within the ironHand project [20]. Previous studies have examined feasibility [20] and the orthotic effect of the ironHand system [21]. In a first randomized controlled clinical study, the effect of prolonged use of such an assisting glove during ADL at home on functional performance of the hand was explored, in comparison to its use as a training tool at home.

## Methods

### Participants

Four sites (1) Roessingh Research and Development (RRD), Enschede, (2) National Foundation for the Elderly (NFE), Bunnik in the Netherlands, (3) Eskilstuna Kommun Vård- och omsorgsförvaltningen (ESK), Eskilstuna in Sweden and (4) terzStiftung (TERZ), Berlingen in Switzerland were involved in the recruitment of the participants. Inclusion criteria for participation into this study were: older adults over the age of 55; self-reported difficulties in performing daily activities, related to hand function decline; at least 10 degrees of active flexion and extension movement of the fingers; able to don/doff the glove by themselves; discharged from specific arm/hand therapy; sufficient cognitive status to understand two-step instructions; (corrected to) normal vision; and living at home. Potential participants were excluded if they had: severe sensory problems, acute pain or wounds on their hands that may create problems when wearing the glove; severe contractures limiting passive range of motion; insufficient knowledge of the Dutch, Swedish or German language to understand the purpose or methods of the study; and participation in other studies that can affect functional performance of upper limb. A power calculation wasn’t applicable for defining the sample size due to the explorative and innovative nature of the study, so the sample size was based on pragmatic grounds. The current findings will serve as input for power calculations for future clinical trials.

An informed consent form was signed by both the participating individuals and the researchers before the study started. The study was approved by the local Medical Ethical Committees in the Netherlands (registration number: NL56746.044.16), Switzerland (registration number: KEKTGOV2015/16) and Sweden (registration numbers: 2016/923-31/2 and 2017/466-32). For this study, recruitment of participants started June 14, 2016 and ended April 11, 2017. The authors confirm that all ongoing and related trials for this drug/intervention are registered.

### Design

**Fig 1:**
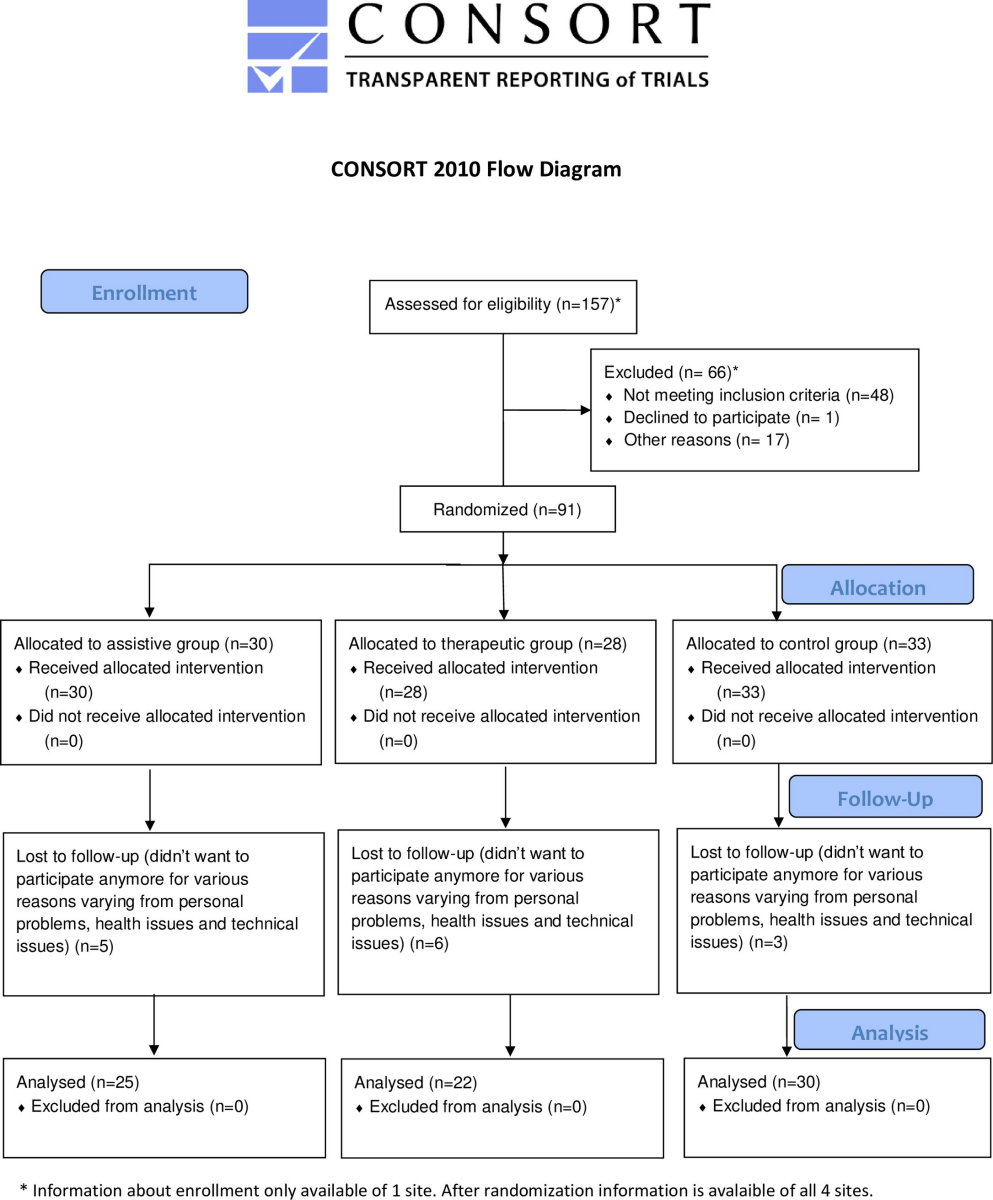
Enrolment participants.

In this multicentre, longitudinal, pilot randomized controlled trial, participants were randomly assigned (using a pre-defined block randomization list, allocating 6 participants per block per center) into three different groups (assistive, therapeutic or control group) (Fig 1). The assistive group used the ironHand system independently during the performance of ADL at home or at work. The therapeutic group performed hand exercises with the ironHand system independently at home and the control group did not receive the ironHand system nor followed any arm/hand therapy. The duration of the treatment was 4 weeks for all groups.

During evaluation sessions within one week before the start of the study and within one week post training, the participants performed various hand function tests to assess the therapeutic effect of the different modes of the ironHand system. All tests were performed in a controlled environment across the four sites: RRD, NFE, ESK and TERZ.

The researchers involved in the study received instructions about how to handle, operate and explain use of the ironHand system to participants by personnel from the technical project partners (Bioservo Technologies AB, Hocoma AG) prior to the start of the study. Also, they were instructed on how to execute the hand function tests by researchers from RRD (coordinator of the study), following a standard procedure.

### Intervention

#### The ironHand assistive group

Participants assigned to the ironHand assistive group received the wearable soft-robotic glove for independent use as an assistive tool during daily activities at home or work for 4 weeks. It was recommended to use the assistive ironHand glove at least 180 min/week during the most common ADL, such as dressing/undressing, eating/drinking, functional transfers and personal hygiene. Nevertheless, they were free to choose for which activities, when and for how long they used the glove. The participants were asked to register the amount of use and activities in which they used the ironHand system in a diary.

#### The ironHand therapeutic group

**Fig 2:**
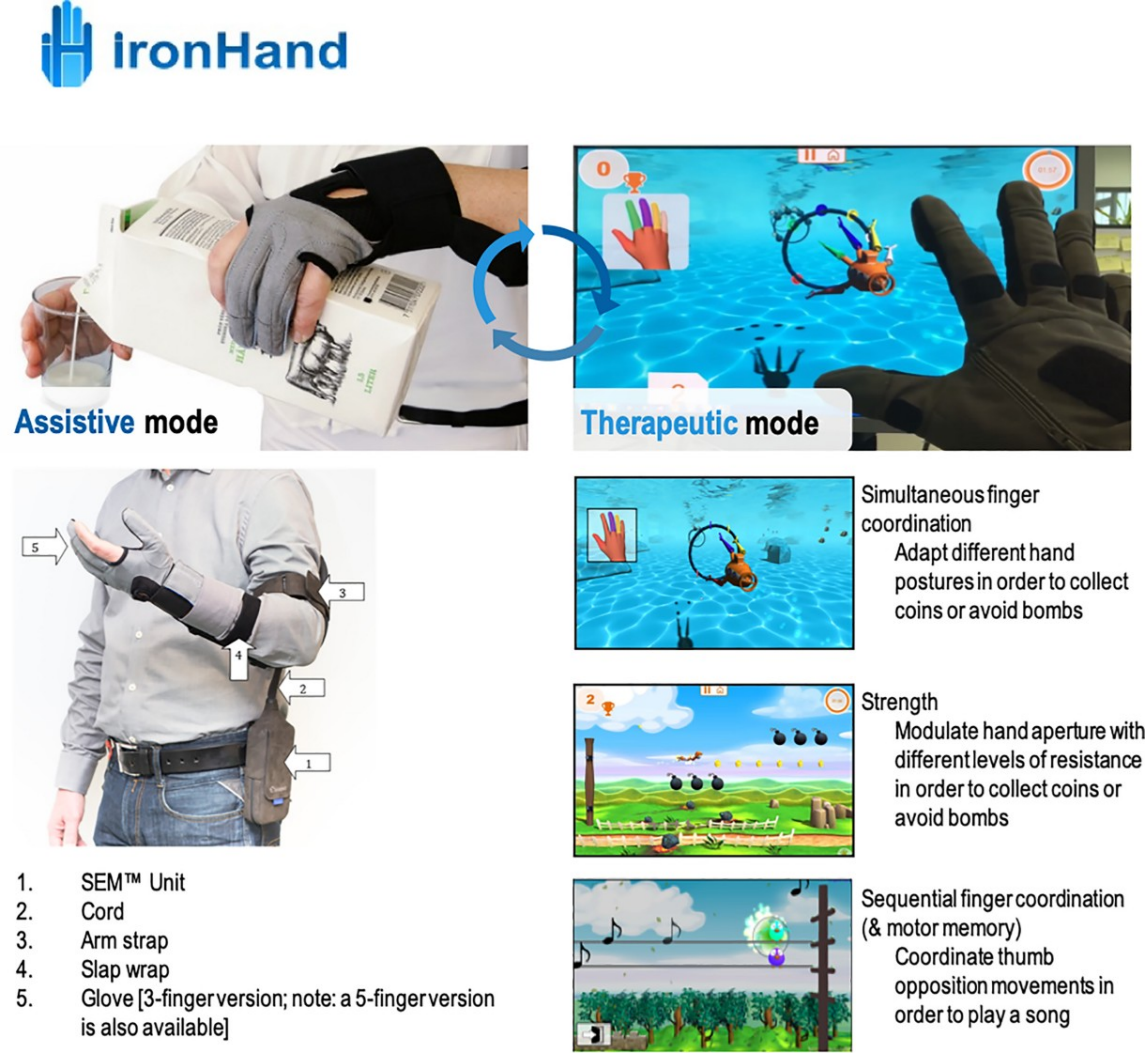
Overview of the ironHand system with assistive functionality (left panel) and therapeutic functionality (right panel). * Reprinted from Bioservo Technologies under a CC BY license, with permission from Bioservo Technologies, original copyright 2017.

Participants assigned to the ironHand therapeutic group used the soft-robotic glove only in combination with a laptop with the therapeutic ironHand software, as a training tool, independently at home for 4 weeks. They were instructed to only use the glove during training exercises with the laptop, they were not allowed to use the glove as an assistive tool during daily tasks. These participants were recommended to perform the hand exercises (games) with the ironHand system for (a minimum of) 180 minutes a week. These exercises were controlled by active arm and hand movements, recorded from flex and force sensors in the glove. Therefore, a calibration procedure presented as a game was performed, to assess participants’ current active range of motion of the hand and fingers at the end of the baseline session. After the calibration game, the therapist made a therapy plan (in which the exercises and starting levels were defined) based on the limitations and treatment goals of the participants. The therapist could choose three different games designed to train hand strength, simultaneous finger coordination and sequential finger coordination (see Fig 2) and three difficulty levels (easy, medium and high). During the exercises, participants received feedback about points collected during the game and corresponding scores. The participants were asked to register the amount of use and games that were played in a diary.

#### The control group

Participants assigned to the control group did not follow a specific intervention during the intervention period. They continued their normal activity pattern of their most-affected hand.

Participants of the assistive and therapeutic group received an ironHand system for their most-affected hand. Before the participants took the ironHand system home, researchers gave instructions about all relevant aspects of the ironHand system, demonstrated it to and practiced with the participant, until the researchers were confident that the participant knew how to use the system at home properly, according to their group allocation (assistive vs. training tool). Additionally, participants received a manual with most important information about the system and a phone number that they could call in case of problems. Furthermore, participants of all three groups were contacted weekly during these 4 weeks of intervention to make sure the participant was doing well and to investigate the progress of the participant. If the games or difficulty level was too easy or too difficult for the participants of the therapeutic group, it was possible to change the order of games and difficulty levels via remote access during their weekly contact.

### Device

The ironHand system was developed to support older adults and patients with self-reported hand function limitations during the performance of daily activities (the assistive functionality) or hand training exercises (therapeutic functionality). The assistive functionality of the ironHand system (see Fig 2, left panel) provides extra strength to the grip of the thumb, middle finger and ring finger of persons with reduced hand function. The grip support is applied by artificial tendons in the wearable soft-robotic glove (placed along the length of the fingers), actuated via motors in the control unit of the system. The extra grip strength is modulated by pressure sensors (Interlink Electronics) in the finger tips. An intention detection logic ensures that the extra strength to the grip is activated in a natural and intuitive way and only after an active contribution by the user. Furthermore, the actuators of the system provide extra force to the grip in proportion to the grip force applied by the user.

The therapeutic functionality of the ironHand system (see Fig 2, right panel) provides a motivating game-like environment to train specific aspects of hand function, such as hand/finger strength, finger coordination or finger independence. The system consists of a therapeutic platform referring to a computing system (e.g. PC or laptop) to which a soft-robotic glove can be connected. The flex sensors along the dorsal side of the 5-finger glove control the hand training exercises played on the PC or laptop. The hand training exercises, assessments, connectivity to other devices, patient database, additional safety mechanisms and user interface are embedded in the therapeutic software.

### Evaluation

For both evaluation sessions, the same order of tests was applied, as follows. First, maximal handgrip strength of the most-affected hand was assessed as primary outcome measure. Subsequently, secondary outcome measures were assessed, starting with pinch strength of the most-affected hand were measured with a dynamometer. Next, hand function performance of the most-affected hand was measured with the Jebsen-Taylor Hand Function Test (JTHFT) and the Box and Blocks Test (BBT). The System Usability Scale (SUS), that measures subjective experience of usability of the ironHand system, was completed at the end of the post-evaluation session, only by the participants of the assistive and therapeutic intervention groups.

### Assessments

#### Maximal handgrip strength

Maximal handgrip strength was measured with the Jamar hydraulic hand dynamometer, Patterson Medical Ltd., Warrenville, IL, USA, with the handle position set at 4 for all attempts for all subjects. The positioning of each participant was standardized as described by the American Society of Hand Therapists [22]. The participant had to squeeze the handgrip of the dynamometer maximally for 5 seconds. Handgrip strength was expressed in kilogram-force (kgf). Each participant had three attempts, with at least 60 seconds of rest between subsequent attempts. The best of three consistent attempts was used for analysis.

#### Maximal pinch strength

Maximal pinch strength was measured with the BaselineLite Hydraulic Pinch Gauge dynamometer (Fabrication Enterprises, White Plains, New York, USA), following the same positioning procedure as described for the maximal handgrip strength test. The pinch strength was measured between the thumb and index finger and thumb and middle finger. The participant was instructed to grasp the pinch dynamometer with the distal segment and ventral side of the thumb and finger. The other fingers were not allowed to give any support. The subject had 3 attempts for each combination and between all the attempts was at least 60 seconds rest. The highest value of the three consistent attempts was used for further analysis.

#### Jebsen-Taylor Hand Function Test (JTHFT)

The JTHFT assesses functional performance and consists of 7 different unilateral hand skill tasks related to ADL: (1) writing 1 sentence of 24 letters (2) turning over 7.6- x 12.7-cm cards (3) picking up small, common objects (i.e., paper clips, coins and bottle caps) and move these to a box (4) simulated feeding (i.e., teaspoon with beans) (5) stacking checkers (test of eye-hand coordination) (6) picking up large empty cans (7) moving weighted (450 g) cans [23, 24]. The subject performed each task with the most-affected hand while sitting comfortably close to the table. The duration of each task from start (lifting hand from table) to completion of the task was recorded in seconds with a stopwatch (maximal duration is 120 seconds per task) and summated as the total score.

#### Box and Blocks Test (BBT)

The BBT evaluates unilateral gross manual dexterity. The subject had to grasp and transport as many blocks as possible within one minute from one compartment to the other, one by one, over a partition. The number of blocks counted after one minute serves as the outcome measure [25, 26].

#### System Usability Scale (SUS)

Subjective experiences of system usability were measured with the SUS. The 10 questions of the SUS were scored on a 5-point Likert scale ranging from 1-strongly disagree till 5-strongly agree. The total score of the SUS ranges from 0–100 and is calculated as described in [27]. A higher score indicates better usability of the system. A system that scores below 50 on the SUS can be almost certain of usability difficulties in the field and is not acceptable, a SUS score between 50–70 indicates marginal acceptability, a SUS score above 70 indicates a good probability of acceptance, a SUS score above 85 indicates excellent usability and a SUS score above 90 indicates best imaginable [28, 29].

Only participants of the assistive and therapeutic group completed the SUS, because the control group did not have sufficient experience with the ironHand system to validly answer questions about its usability.

#### Use time

Use time was recorded using a diary in both assistive and therapeutic intervention groups.

### Statistical analysis

Statistical analyses were performed with the software package IBM SPSS statistics version 23.0 for Windows. First, histogram plots of all outcome measures were checked for normal distribution by visual inspection. Descriptive statistics, using mean ± standard error of the mean (SEM) or the median (interquartile range), were used to describe the participants’ characteristics, outcome measures and use time. To confirm equality of characteristics across groups after randomization, an One-Way ANOVA was performed for ratio/interval data and a Chi-squared test or the Fisher exact test for nominal/ordinal data.

To investigate the training effect of ironHand system use, a mixed-model analysis was performed for each outcome measure (handgrip strength, pinch strength, BBT, JTHFT), with time of measurement as within-subject factor and group as between-subject factor, including its interaction (time x group) to assess whether there is a difference in treatment effect over time between groups. For the JTHFT, first a log-transformation was performed before the mixed-model analysis was performed, to normalize the data, which was successful for all groups on the following subtasks of the JTHFT: ‘card turning’, ‘checkers’, ‘large, heavy objects’, and total performance time JTHFT. For the JTHFT subtasks of the groups that did not follow a normal distribution, even after a log-transformation, (‘writing’ therapeutic group, ‘small, common objects’ assistive group, ‘simulated feeding’ assistive and control group, ‘large, light objects’ assistive group, ‘total performance time JTHFT–without subtask writing’ assistive and control group), a Wilcoxon signed rank test was performed to compare pre-post evaluation. Thereafter, the individual differences between pre-post evaluation for these JTHFT subtasks were calculated for each group, which did follow a normal distribution. Subsequently, a one-way ANOVA was performed for these subtasks to investigate the difference between groups for training effect of ironHand system use. The difference in amount of ironHand use between the assistive and therapeutic group was analysed with the Mann-Whitney U test. Additionally, correlation analyses were performed for ironHand use time with differences between pre-post evaluations of all clinical assessments using the Spearman’s correlation coefficient. The overall significance level was set at α≤0.05.

## Results

A total of 91 participants (Table 1) were included in the study, of which 14 participants dropped out (for various reasons varying from personal problems, health issues and technical issues). Of the 91 included participants, 74 (81%) were older adults with self-perceived hand function limitations due to various age-related problems (of which the most prominent were rheumatoid arthritis and osteoarthritis), and 17 (19%) were stroke patients with hand function limitations. Of those 91 participants, 30 were allocated to the assistive group (5 dropped out), 28 to the therapeutic group (6 dropped out) and 33 to the control group (3 dropped out) (see Fig 1). When using baseline handgrip strength as indicator for self-reported mobility difficulties (<37 kg (men) and <21 kg (women) [30]), the current participants comprised largely people classified with weak grip (95%).

**Table 1:** Descriptive characteristics of participants (N = 91)^a^.

Characteristic	Total (n = 91)	Assistive group (n = 30)	Therapeutic group (n = 28)	Control group (n = 33)
Age (years)^b^	73 (±1)	74 (±2)	71 (±2)	73 (±1)
Gender (M/F)^b^	28 (31%) / 63 (69%)	10 (33%) / 20 (67%)	5 (18%) / 23 (82%)	13 (39%) / 20 (61%)
Dominant hand (R/L)^b^	83 (91%) / 8 (9%)	27 (90%) / 3 (10%)	24 (86%) / 4 (14%)	32 (97%) / 1 (3%)
Most-affected hand (R/L/both)^b^	59 (65%) / 18 (20%) / 14 (15%)	20 (67%) / 6 (20%) / 4 (13%)	15 (54%) / 7 (25%) / 6 (21%)	24 (73%) / 5 (15%) / 4 (12%)
Baseline Handgrip strength (kgf)^b^	15.3 (±0.8)	16.3 (±1.4)	12.4 (±1.5)	16.9 (±1.3)

^a^Mean (±SEM) or Count (%)

^b^no significant difference between groups (p≥0.053)

### Therapeutic effect of the ironHand system

Overall, inspection of Table 2 showed that participants of the assistive, therapeutic and control group improved performance on almost all outcome measures after 4 weeks, which is most pronounced in the therapeutic group.

**Table 2:** Scores for all intervention groups of handgrip strength, pinch strength and BBT^a^.

	Assistive group		Therapeutic group		Control group	
	Pre	Post	Pre	Post	Pre	Post
Handgrip strength	16.3 ± 1.5	18.1 ± 1.5	12.4 ± 1.1	15.4 ± 1.2	16.9 ± 1.5	16.6 ± 1.5
Pinch strength–thumb and index finger	3.5 ± 0.3	3.7 ± 0.3	3.0 ± 0.3	3.4 ± 0.3	3.3 ± 0.3	3.5 ± 0.3
Pinch strength–thumb and middle finger	3.1 ± 0.3	3.1 ± 0.3	2.5 ± 0.3	2.9 ± 0.3	2.5 ± 0.3	2.8 ± 0.3
BBT	43.2 ± 2.8	46.8 ± 2.9	42.9 ± 2.9	45.4 ± 3.0	43.9 ± 2.7	46.5 ± 2.7

^a^Data is represented as mean ± SEM

Handgrip strength and pinch strength (of thumb with index finger) only increased significantly (p≤0.039) in the therapeutic group by respectively 3.0 kgf (24.9%) and 0.4 kgf (14.4%) from pre- to post-evaluation (see Table 2). The number of blocks transferred during the BBT only increased significantly in the assistive (3.6 blocks, 8.5%, p = 0.007) and control group (2.6 blocks, 5.9%, p = 0.025) from pre- to post-evaluation, indicating better performance (see Table 2).

Results (mean ± SEM) of the different subtasks of the JTHFT are presented in Table 3. Lower performance time on any subtask indicates better performance. The assistive group improved performance (p≤0.015) in 5 subtasks of the JTHFT (‘card turning’, ‘small, common objects’, ‘simulated feeding’, ‘large, light objects’, ‘large, heavy objects’), the therapeutic group improved performance (p≤0.029) in 4 subtasks of the JTHFT (‘card turning’, ‘small, common objects’, ‘checkers’ and ‘large, heavy objects’) and the control group improved performance (p≤0.017) in 5 subtasks of the JTHFT (‘writing’, ‘card turning’, ‘small, common objects’, ‘large, light objects’ and ‘large, heavy objects’) after 4 weeks intervention.

**Table 3:** Subtasks scores for all intervention groups on the JTHFT^a^.

Subtask	Assistive group		Therapeutic group		Control group	
	Pre	Post	Pre	Post	Pre	Post
Writing^b^	23.1 ± 1.1	22.2 ± 1.1	18.9 (14.5–38.0)	15.7 (12.3–32.8)	21.3 ± 1.1	18.7 ± 1.1*
Card turning	11.3 ± 1.1	8.8 ± 1.1*	12.2 ± 1.1	9.3 ± 1.1*	10.3 ± 1.1	8.4 ± 1.1*
Small, common objects	10.5 (8.4–21.0)	9.6 (7.3–12.5)*	13.2 ± 1.1	11.4 ± 1.1*	12.1 ± 1.1	10.8 ± 1.1*
Simulated feeding	11.9 (8.8–16.5)	10.0 (8.5–14.5)*	12.7 ± 1.1	11.7 ± 1.1	10.2 (7.7–15.0)^b^	10.2 (7.8–14.1)
Checkers	10.1 ± 1.2	9.7 ± 1.2	10.0 ± 1.1	8.3 ± 1.1*	8.0 ± 1.1	7.6 ± 1.1
Large, light objects	6.6 (5.2–8.7)	5.4 (3.9–7.1)*	7.2 ± 1.1	6.4 ± 1.1	6.9 ± 1.1	6.2 ± 1.1*
Large, heavy objects	7.9 ± 1.1	6.1 ± 1.1*	7.7 ± 1.1	6.2 ± 1.1*	7.6 ± 1.1	6.6 ± 1.1*
Total performance time^b^	80.7 ± 1.1	70.0 ± 1.1*	82.9 ± 1.1	71.8 ± 1.1*	73.6 ± 1.1	66.3 ± 1.1*
Total performance time–without subtask writing	55.8 (45.0–94.2)	46.2 (35.3–62.2)*	64.6 ± 1.1	54.6 ± 1.1*	52.0 (39.4–71.8)	47.5 (37.2–67.5)*

^a^Normally distributed data is represented as mean ± SEM and data not following the normal distribution is represented as median (interquartile range)

^b^Missing data of 5 participants in the assistive group, 6 in the therapeutic group and 7 in the control group because they were not able to perform the writing task

*significant difference from pre-evaluation.

In most outcome measures (handgrip and pinch strength, BBT and JTHFT), the improvement over time did not differ significantly between groups (p≥0.221). For handgrip strength an interaction effect for group and time (p = 0.009) was present, with improvements from pre- to post evaluations in the therapeutic group (p<0.001), but no change in the assistive group (p = 0.135) and control group (p = 0.561). When represented as relative change with respect to baseline values, the therapeutic group became 25% stronger after 4 weeks of ironHand system use, in contrast to an improvement in the assistive group of 12% and a decrease of 2% in the control group.

### Use time

**Fig 3:**
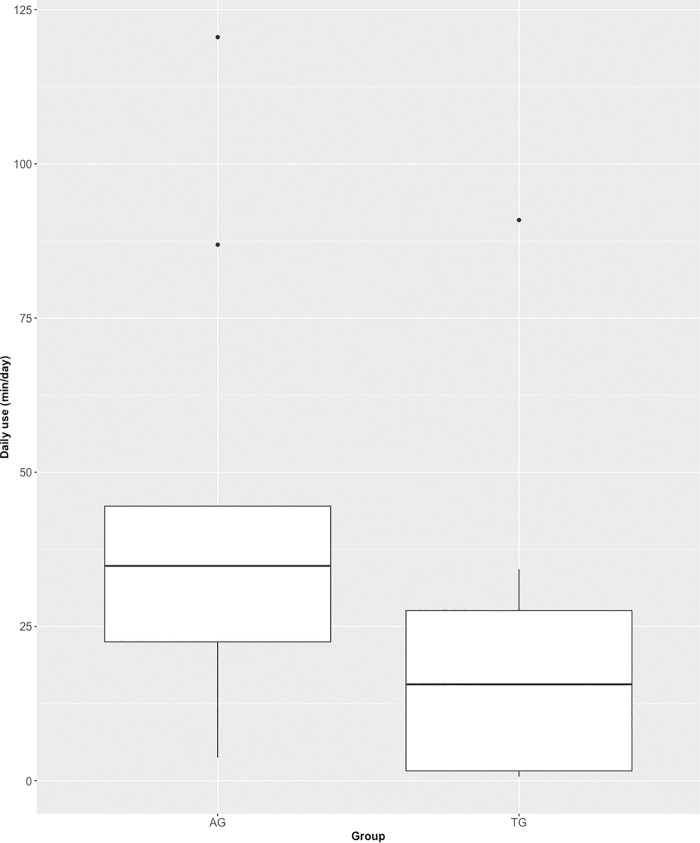
Daily use (min/day), separately for assistive group (AG) and therapeutic group (TG).

Use time was reported through diaries, with complete diaries available from 21 participants (out of 77 participants). Total use time of these participants was on average 879 (±194) minutes, or 15 (±3) hours. When calculated as average use time per day, this reflects 31 (±7) minutes each day for 4 weeks. The mean training duration, averaged per week over 4 weeks, was 220 (±49) minutes. When distinguishing between intervention groups (Fig 3), we can observe that the assistive group (n = 9) used the system on average about twice as long as the therapeutic group (n = 12) (average daily use 45 ± 12 min vs. 21 ± 7 min). This observed group difference was significant (p = 0.033). However, it should be noted that the variation between individuals is large, ranging from 18 to 3375 minutes per 4 weeks.

Correlation analyses showed for some outcomes a relation between use time and change scores per group, for the assistive and therapeutic groups. Most pronounced correlations, approaching significance, were observed for the assistive group with pinch strength between thumb and middle finger (ρ = 0.67, p = 0.071) and the therapeutic group with pinch strength between thumb and index finger (ρ = 0.55, p = 0.062).

### Experiences of end-users

SUS data were available from participants allocated to the assistive or therapeutic groups (total 58 included, of which 47 completed the post-evaluation and 11 dropped out).

**Fig 4:**
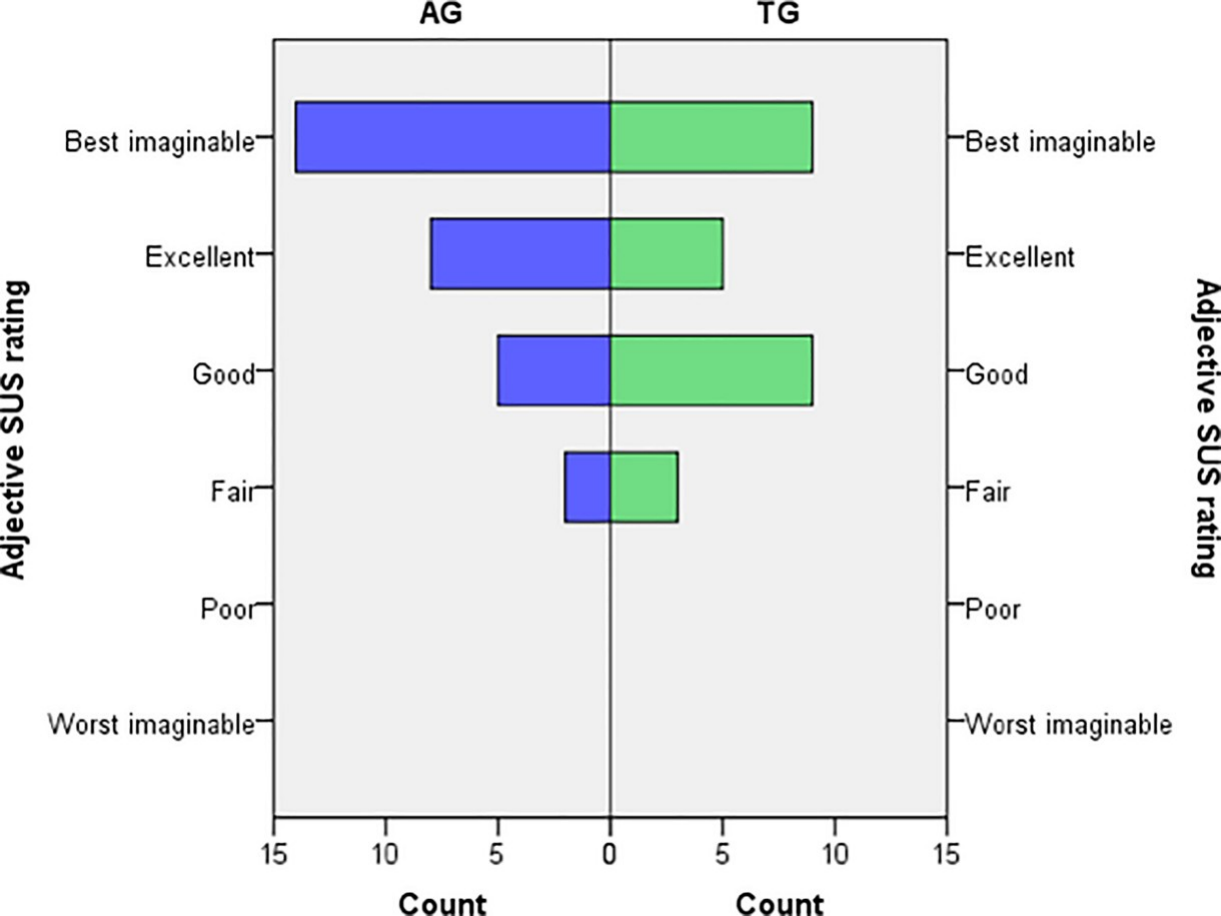
Frequency distribution of SUS score, categorised by adjective ratings [29], separately for assistive group (AG) and therapeutic group (TG).

Mean SUS score across both groups was 73 (±2), varying on individual level between 48 and 100. When divided per group, mean SUS for the assistive group (n = 25) was 77 (±3) and for the therapeutic group (n = 22) 69 (±3). According to adjective ratings scales corresponding with the SUS score [29], the ironHand system used as assistive device is perceived as having good usability and used as training tool to have OK usability (Fig 4).

## Discussion

This study shows first of all that older adults that used the ironHand system as assistive device or as a training tool were capable to use the ironHand system by themselves at home. Both groups used the ironHand system for a substantial amount of time, with the assistive group using the ironHand system twice as long as the therapeutic group. Usability of both systems was perceived acceptable. When comparing pre- and post-evaluations for all groups separately, participants of the therapeutic group showed improved unsupported handgrip strength and pinch strength after 4 weeks of ironHand system use, while the improvement in functional performance in the assistive and therapeutic groups did not differ from that in the control group. Only handgrip strength improvements from pre- to post-evaluations did differ between groups, participants of the therapeutic group improved more compared to the assistive and control group. No significant correlation was found between the total duration of ironHand use and changes in performance, for the assistive and therapeutic groups.

To our knowledge, this is one of the first user trials that applied and tested a fully wearable robotic system to support hand function at home for unsupervised use during an extended period of multiple weeks. Moreover, this was done in a large group of older adults with hand function limitations. It also involved two scenarios that provide a unique approach, where the ironHand system was used as assistive device or as a training tool. The findings emerging from this extensive user trial indicate first of all that the ironHand system was very well accepted by the users. The majority of SUS ratings fell in the higher categories: ‘good’, ‘excellent’ and even ‘best imaginable’, for both assistive and therapeutic systems. The findings regarding user experiences showed an improved usability across the subsequent iterations within the ironHand project, as indicated by a gradual increase of SUS scores of both the assistive system and the therapeutic system across its previous development stages [20, 21, 31]. The present level of usability indicates high probability for acceptance in the field [28].

The usability of the ironHand system regarding SUS scores is similar to that reported for other assistive or rehabilitation technology. A passive orthosis to support wrist and hand movements of stroke patients during game-like exercises at home received an average SUS score of 69 in its first iteration [32] and 73 in its second iteration [33]. The usability ratings of the ironHand system are comparable to these findings, with an average SUS score of 73. In terms of use time, the ironHand system scored considerably better (220 vs. 105 and 118 minutes a week) than the passive orthosis reported by Nijenhuis et al. [32, 33]. In addition, the study of Wittmann et al. 2016 [34] showed promising results on feasibility of high dose unsupervised arm therapy at home for stroke patients using an inertial measurement unit-based virtual reality system.

The findings from this study showed that unsupported handgrip strength improved in the therapeutic group and functional task performance improved after 4-week use of a soft-robotic glove at home, either as assistive device or as training tool. However, improvement of functional performance in the technology-assisted groups was not different from the improvement observed in the control group. Remarkably, the control group received no exercise programme and still improved their performance. It is possible that participants improved their performance due to a learning effect on the clinical tests. Such a learning effect might be even more pronounced in those participants that used their non-dominant hand. In addition, a role of response bias (in particular, demand characteristics [35]) cannot be excluded either. It is possible that participants allocated to the control group became more aware of their affected arm in daily life due to their participation to the study in itself, and used their affected arm/hand more than they would have done prior to the study, even though they were instructed to continue their normal activity pattern. Unfortunately, we did not measured their normal arm activity in daily life, so we could not control for this. These issues make it difficult to explain the observed improvement in the control group in the current study, which complicates interpretation of the current findings.

Nevertheless, hand strength did improve more after therapeutic use of the ironHand system than in the assistive or control group. In terms of application as a rehabilitation tool in the therapeutic group, improvements in handgrip strength, pinch strength and functional use of the arm/hand have been reported after robotic hand rehabilitation, applying mostly stationary and/or portable systems as training device in clinical settings after stroke [36–38]. The study of Vanogli et al. 2017 [38] showed that improvements after robotic rehabilitation were significantly higher to those after control intervention consisting of a dedicated program of regular exercises. The studies of Nijenhuis et al. [32, 33] examined the effect of a portable, though not wearable, hand training system after stroke, applied at home with offline supervision, and also reported improved hand function. In this case, the system was stationary yet portable, and not suitable for assistance of daily life activities. In comparison to a control group receiving regular home exercises, no difference was found [39], indicating that technology-assisted training results in similar improvements in hand function as regular exercise programmes. Several wearable systems for hand rehabilitation have been developed recently (see [12] for an overview), but no other clinical studies focusing on hand function changes using fully wearable systems intended for home use could be identified at this point, especially in comparison to use of the wearable system as daily support and/or a control group.

Regarding training interventions for reduction of age-related decline in hand function, intensive resistance training has been reported as one of the most efficient interventions to counter or prevent sarcopenia, with improvements of up to >50% in strength after six weeks of training at a rhythm of 2–3 sessions per week [40]. After ironHand therapeutic use, which partially involved resistance training exercises, substantial improvements in handgrip strength were observed, indicating that such application of a soft-robotic glove combined with dedicated (strength) exercises may be a way to provide motivating strength training to address sarcopenia.

Remarkably, a similar effect, although to a smaller degree, was present in older adults using the assistive system to support performance of functional activities during daily life. Moreover, there was no decline in strength and functional performance after using the assistive system. This suggests that using the hands intensively during submaximal yet highly functional activities has a training effect in itself, and could be beneficial in dealing with sarcopenia as well, although the comparable findings in the control group complicate this inference. A review indicating that a multi-component physical exercise programs (involving endurance, flexibility, strength, etc.) showed less functional decline in frail elderly than single-component programs focussing on strength only [41], may indicate support for this. This may also be relevant specifically in case of hand osteoarthritis, where exercise has shown to have a beneficial influence on hand function, pain and finger stiffness [42]. Similarly, functional practice is known as one of the essential elements for motor relearning after stroke [6], suggesting that application of a soft wearable robotic glove may be a dedicated solution to enable highly functional treatment. It is also possible that the support from the assistive glove enabled people to use their arms and hands in more strenuous and/or higher dose activities than would otherwise be possible in their daily life. Considering that low physical activity and low amounts of exercise seem to be the most powerful predictors of ADL disability [43], technological support as proposed with this ironHand system facilitating people to increase their activity level during their daily lives, can be very promising to counter or prevent functional decline associated with ageing.

Recently, development of wearable soft-robotic gloves aimed at supporting people with disabilities in daily life have attained considerable attention [13–18, 44], and the field is growing [45]. To our knowledge, none of these research groups have yet investigated the effect of such assistive robotic devices in daily life among a large sample of people with hand function limitations. One research group has published a study into the direct influence of their soft-robotic glove, pneumatically actuated and controlled by muscle activity [46]. So far, they showed a reduced performance on JTHFT in one healthy subject compared to normative JTHFT data of healthy subjects [13] and increased performance on the BBT with the assistive glove in one patient with muscular dystrophy [47]. The results of the JTHFT are in line with those from the ironHand project (as reported in [21]), but the findings on the BBT are not in line with those from the ironHand project (as reported in [21]). Other wearable systems for hand rehabilitation have been developed as well [14, 44, 46], but these have neither been evaluated during a longitudinal study.

These recent technological developments in the field of wearable robotics are directed towards more unobtrusive support of the arm in a real life setting, which is intended to further facilitate highly functional and intensive training, even enabling more self-administered scenarios [37]. This presents a dual application of soft-robotics: as assistive device in daily life, which is believed to have a direct benefit on functional independence [13, 14] and/or as training tool to improve unsupported functioning. The findings of the orthotic effect of the ironHand assistive system [21, 48] and the training effect of the ironHand therapeutic system (as described in this study) support that both of these approaches can indeed benefit people with hand function limitations. Even more, based on the current findings a third scenario appeared, where users of the assistive system improve unsupported hand function over time. This may be related to a large variety of functional activities being supported in daily life throughout the day, which likely turns everyday activities into highly functional and intensive practice. This underlines the major potential that a soft-robotic wearable glove system can have for increasing functional independence of a very large group of users with varying causes of hand function limitations.

Even though the current study is one of its kind and has generated a large amount of highly valuable information regarding the user acceptance and potential impact on daily lives of the ironHand system, some aspects of the study have given rise to several lessons and recommendations. First, timed performance of functional tasks may not necessarily capture the full potential of the training effect provided by the ironHand system. The JTHFT was specifically selected based on its validity for application in the elderly population, and we anticipated that a timed outcome measure would be more sensitive to change in a relatively high functioning population (participants were living at home independently and were able to perform most ADL tasks, despite perceiving limitations in this respect). Adding an outcome measure that can capture more detailed information about movement or task execution in future research should be considered to better understand how a wearable glove system influences hand function. Second, the population involved in the current trial was rather heterogeneous, as intended to examine the potential of the ironHand system across a broad user population. A more in-depth analysis investigating potential differences between subgroups according to disorder or level of physical limitations could shed more light on the therapeutic potential of the ironHand system, and might distinguish which users would benefit most from assistive or therapeutic application of the ironHand system. Third, not only participants in the assistive and therapeutic group showed improvements in performance but also the participants in the control group showed improvements in performance on some outcome measures, for example on the BBT. This indicates that there is also a learning effect for performing the clinical tests. Fourth, in each center detailed instructions were given about the recruitment and inclusion procedure, explaining carefully that allocation to groups should strictly follow the randomization list in order of inclusion. Nevertheless, potential influence of selection bias due to treatment foreknowledge can’t be controlled or ruled out in this study. Fifth, the current study results might be biased due to the awareness of the intervention group by the participant and the assessor or the fact that some variables were only assessed on a very limited proportion of the overall sample size. It is not possible to compare the current results of this study with other studies. Therefore, more research is needed.

## Conclusion

The current pilot randomized controlled trial is one of the first of its kind in investigating the impact of using a wearable soft-robotic device for hand support during multiple weeks at home. Findings of this user trial showed that participants were capable to use both modalities (assistive and therapeutic) of the ironHand system by themselves for a substantial amount of time at home. After 4 weeks of ironHand use in the therapeutic group, participants showed improvements in unsupported handgrip strength and pinch strength. Functional performance improved not only after 4 weeks of ironHand use (assistive and therapeutic), but also in the control group who received no additional exercise or treatment. Only handgrip strength improved more in the therapeutic group compared to the assistive and control group. These results suggest that, even though the findings from the control group complicate interpretation of the results on functional performance from the technology-supported groups, technological support from wearable robotics as provided through the ironHand system may be promising to improve functional performance in persons with hand problems associated with ageing in general, or specific age-related disorders (varying from rheumatoid arthritis and osteoarthritis to stroke).

## Supporting information

S1 Checklist (DOC)

S1 Dataset (XLSX)

S1 Protocol (PDF)
