# Supplementary material for: Home rehabilitation supported by a wearable soft-robotic device for improving hand function in older adults: A pilot randomized controlled trial
Source: PLoS One. 2019 Aug 6;14(8):e0220544. doi: 10.1371/journal.pone.0220544 (PMC6684161; doi:10.1371/journal.pone.0220544)
Supplement: S1 Protocol — (PDF) [file pone.0220544.s003.pdf]

PROTOCOL SIGNATURE SHEET

Name	Signature	Date
**Sponsor or legal representative:**<br>Roessingh Research & Development BV, Enschede<br><br>**Head of Department:**<br>M. Luizink, ir.<br>Director Roessingh Research & Development BV, Enschede		12 mei '16
**[Coordinating Investigator/Project leader/Principal Investigator]:**<br>G.B. Prange-Lasonder, PhD<br>Researcher Roessingh Research & Development BV, Enschede		12 mei '16

The ironHand system integrates both support in ADL and functional training exercises to improve impaired hand function

iH_TS[56746]_v2

PROTOCOL TITLE 'The ironHand system integrates both support in ADL and functional training exercises to improve impaired hand function'

**Protocol ID**	**iH_TS[56746]_v2**
**Short title**	**A robotic glove that supports ADL and therapeutic exercises**
**Version**	**2.0**
**Date**	**09-05-2016**
**Coordinating investigator/project leader**	G.B. Prange-Lasonder, PhD<br>Roessingh Research & Development, Enschede<br>Roessinghsbleekweg 33B, 7522 AH Enschede<br>g.prange@rrd.nl; tel: +31 (0)53 487 5759
**Principal investigator(s) (in Dutch: hoofdonderzoeker/ uitvoerder)**	B. Radder, MSc<br>Roessingh Research & Development<br>b.radder@rrd.nl; tel: +31 (0)53 487 5756<br>L. Gaasbeek<br>National Foundation for the Elderly<br>l.gaasbeek@ouderfonds.nl; tel +31(0)30 656 7774
**Sponsor (in Dutch: verrichter/opdrachtgever)**	M. Luizink, ir<br>Roessingh Research & Development BV, Enschede<br>Roessinghsbleekweg 33B, 7522 AH Enschede<br>m.luizink@rrd.nl; tel: +31 (0)53 487 5777
**Subsidising party**	The ironHand project is partly funded by the Ambient Assisted Living Joint Program Call 6
**Independent expert (s)**	M.F. Nederhand, MD, PhD<br>Rehabilitation physician Roessingh rehabilitation centre<br>m.nederhand@rrd.nl; tel: +31 (0)53 487 5705
**Laboratory sites**	Roessingh Research & Development BV<br>Roessinghsbleekweg 33B<br>7522 AH Enschede<br><br>National Foundation for the Elderly<br>Regulierenring 2D<br>3981 LB Bunnik

PROTOCOL SIGNATURE SHEET

Name	Signature	Date
**Sponsor or legal representative:**<br>Roessingh Research & Development BV, Enschede<br><br>**Head of Department:**<br>M. Luizink, ir.<br>Director Roessingh Research & Development BV, Enschede		
**[Coordinating Investigator/Project leader/Principal Investigator]:**<br>G.B. Prange-Lasonder, PhD<br>Researcher Roessingh Research & Development BV, Enschede		

TABLE OF CONTENTS

1. INTRODUCTION AND RATIONALE 10
2. OBJECTIVES 12
3. STUDY DESIGN 13
4. STUDY POPULATION 15
4.1 Population (base) 15
4.2 Inclusion criteria 15
4.3 Exclusion criteria 16
4.4 Sample size calculation 16
5. TREATMENT OF SUBJECTS 17
5.1 Investigational product/treatment 17
5.2 Use of co-intervention (if applicable) 19
5.3 Escape medication (if applicable) 19
6. INVESTIGATIONAL PRODUCT 20
6.1 Name and description of investigational product(s) 20
6.2 Summary of findings from non-clinical studies 22
6.3 Summary of findings from clinical studies 22
6.4 Summary of known and potential risks and benefits 25
6.5 Description and justification of route of administration and dosage 25
6.6 Dosages, dosage modifications and method of administration 26
6.7 Preparation and labelling of Investigational Medicinal Product 26
6.8 Drug accountability 26
7. NON-INVESTIGATIONAL PRODUCT 27
8. METHODS 28
8.1 Study parameters/endpoints 28
8.1.1 Main study parameter/endpoint 28
8.1.2 Secondary study parameters/endpoints (if applicable) 28
8.1.3 Other study parameters (if applicable) 28
8.2 Randomisation, blinding and treatment allocation 29
8.3 Study procedures 29
8.4 Withdrawal of individual subjects 32
8.4.1 Specific criteria for withdrawal (if applicable) 32
8.5 Replacement of individual subjects after withdrawal 33
8.6 Follow-up of subjects withdrawn from treatment 33
8.7 Premature termination of the study 33
9. SAFETY REPORTING 34
9.1 Temporary halt for reasons of subject safety 34
9.2 AEs, SAEs and SUSARs 34
9.2.1 Adverse events (AEs) 34
9.2.2 Serious adverse events (SAEs) 34
9.2.3 Suspected unexpected serious adverse reactions (SUSARs) 35
9.3 Annual safety report 35

9.4 Follow-up of adverse events 35
9.5 [Data Safety Monitoring Board (DSMB) / Safety Committee] 35
10. STATISTICAL ANALYSIS 36
10.1 Primary study parameter(s) and secondary study parameter(s) 36
10.2 Other study parameters 36
10.3 Interim analysis (if applicable) 36
11. ETHICAL CONSIDERATIONS 37
11.1 Regulation statement 37
11.2 Recruitment and consent 37
11.3 Objection by minors or incapacitated subjects (if applicable) 37
11.4 Benefits and risks assessment, group relatedness 37
11.5 Compensation for injury 38
11.6 Incentives (if applicable) 38
12. ADMINISTRATIVE ASPECTS, MONITORING AND PUBLICATION 39
12.1 Handling and storage of data and documents 39
12.2 Monitoring and Quality Assurance 39
12.3 Amendments 39
12.4 Annual progress report 39
12.5 Temporary halt and (prematurely) end of study report 40
12.6 Public disclosure and publication policy 40
13. STRUCTURED RISK ANALYSIS 41
14. REFERENCES 42
15. Appendix 1 45
15.1 Subject characteristics part 1 45
15.2 Subject characteristics part 2 46
16. Appendix 2 47
16.1 Jebsen-Taylor Hand Function test 47
17. Appendix 3 48
17.1 Box and Blocks Test 48
18. Appendix 4 49
18.1 Motor Activity Log (MAL 49
18.1.1 Score formulier 51
18.1.2 Extra aantekeningen voor bij het scoren 52
19. Appendix 5 53
19.1 SF-36 53
20. Appendix 6 56
20.1 System Usability Scale 56
21. Appendix 7 57
21.1 Intrinsic Motivation Inventory 57
22. Appendix 8 59
22.1 Semi-structured interview about user experience – guide 59
22.1.1 Therapeutic Exercises .... **Error! Bookmark not defined.**
22.1.2 Assistive System .... **Error! Bookmark not defined.**

LIST OF ABBREVIATIONS AND RELEVANT DEFINITIONS

ABR	ABR form, General Assessment and Registration form, is the application form that is required for submission to the accredited Ethics Committee (In Dutch, ABR = Algemene Beoordeling en Registratie)
ADL	Activities of daily living
AE	Adverse Event
AR	Adverse Reaction
AS	Assistive system
CA	Competent Authority
CCMO	Central Committee on Research Involving Human Subjects; in Dutch: Centrale Commissie Mensgebonden Onderzoek
CV	Curriculum Vitae
DSMB	Data Safety Monitoring Board
EU	European Union
EudraCT	European drug regulatory affairs Clinical Trials
GCP	Good Clinical Practice
IB	Investigator’s Brochure
IC	Informed Consent
iH	ironHand
IMP	Investigational Medicinal Product
IMPD	Investigational Medicinal Product Dossier
METC	Medical research ethics committee (MREC); in Dutch: medisch ethische toetsing commissie (METC)
NFE	National Foundation for the Elderly
QoL	Quality of Life
RRD	Roessingh Research and Development
(S)AE	(Serious) Adverse Event
SPC	Summary of Product Characteristics (in Dutch: officiële productinfomatie IB1-tekst)
Sponsor	The sponsor is the party that commissions the organisation or performance of the research, for example a pharmaceutical company, academic hospital, scientific organisation or investigator. A party that provides funding for a study but does not commission it is not regarded as the sponsor, but referred to as a subsidising party.
SUSAR	Suspected Unexpected Serious Adverse Reaction

**TS**	**Therapeutic system**
**Wbp**	**Personal Data Protection Act (in Dutch: Wet Bescherming Persoonsgevens)**
**WMO**	**Medical Research Involving Human Subjects Act (in Dutch: Wet Medisch-wetenschappelijk Onderzoek met Mensen**

SUMMARY

Rationale: Elderly people and patients with acute (e.g. stroke) or chronic (e.g. arthritis) diseases frequently experience difficulties in performing activities of daily living (ADL) due to a decline in hand function. They often need personal and/or assistive devices to carry out ADL. However, personal assistance will not result in more independence in performing ADL while assistive devices have the potential to provide the assistance that is necessary to perform ADL independently. New technological innovations can support the functional performance of the arms and hands directly by a wearable soft robotic device assisting a person's own function. By integrating both an assistive robotic device with exercise training, performance of ADL can be enhanced directly and/or via an improved arm and hand function after prolonged use of the hands.

Objective: The primary objective of the present study is to examine the orthotic and therapeutic effect of the ironHand (iH) system, consisting of both an assistive and therapeutic module, by elderly and diagnosed patients with hand function problems, after using the iH system for a four weeks training period at home. Secondary objectives are related to user acceptance, including usability, satisfaction, motivation and compliance.

Study design: A randomized controlled trial design will be conducted, in which both the elderly and patient population will be randomized into three groups; the iH assistive group, the iH therapeutic group and the control group. Evaluation is based on one baseline measurement and one evaluation measurement within one week after the intervention period of four weeks.

Study population: In total, twenty-seven elderly and fifteen stroke patients with an age over 55 years, will participate in this study.

Intervention (if applicable): The intervention period for all three groups will last for a period of four weeks. The iH assistive group will use the wearable robotic device during ADL at home and the therapeutic iH group will use the wearable robotic device as a training tool using games via the patient user interface. Participants of the control group do not follow an intervention program. In the iH assistive group, participants are recommended to use the wearable robotic device for 180 minutes a week during ADL at home. The participants in the iH therapeutic group are recommended to train the hand 3 times a week for 60 minutes by performing game exercises while wearing the robotic device to support hand opening and strength and to control the game exercises on a screen. During the four weeks intervention period, all three groups will be monitored by a therapist.

Main study parameters/endpoints: Jebsen-Taylor Hand Function Test

Nature and extent of the burden and risks associated with participation, benefit and group relatedness: The iH system may have a beneficial effect on hand function, by directly improving functional task performance or by using it as a training tool. It may be possible that

the functional use of the hand improves, allowing people to be more active in ADL and to maintain or improve their health status. However, the exact benefit cannot be predicted, because this is the topic of the current research.

The risks for the subjects are limited to a minimum. The iH system is a device that facilitates hand grip and opening as initiated by the user him/herself. It provides support only when necessary based on voluntary, active initiation by the person him/herself. Furthermore, the iH system is a so-called soft-robotics device, constructed from soft materials that are comfortable to wear and compliant with human movement. This prevents potential occurrence of pressure points for example. All movements conducted during the study will consist of arm/hand movements that normally occur in ADL and within the abilities of each individual. Additionally, all the evaluation measurements used in these studies are non-invasive and involve no risks for the subjects.

1. INTRODUCTION AND RATIONALE

The hand is important to perform activities of daily living (ADL). However, many people experience a loss of hand function as result of acute diseases (e.g., stroke), chronic diseases (e.g., arthritis) or ageing. The experience of a decline in hand function can be a result of a reduction in grip strength, finger dexterity, sensation or muscle coordination [1-3]. As a consequence, people with a loss of hand function have difficulties with holding and manipulating objects [4], subsequently leading to difficulties with independently performing ADL [5-7]. These limitations can have a negative effect on their participation in society or quality of life [8-11]. Therefore, it is important to restore and maintain hand motor function of people with an impaired hand function.

Different approaches can be used to improve independence in ADL, such as personal assistance, assistive devices or hand exercise training. In contrast to personal assistance, assistive devices and hand exercise training may result in increased independence in performing ADL without relying on others.

Assistive devices have the potential to provide the assistance that is necessary to perform independently ADL [12]. There are many assistive devices available that people can use to compensate for the loss of functionality in their upper limb motor function [13, 14]. These assistive devices can range from simple assistive tools (e.g. knife with an adapted handle) to fully robotic systems that can substitute activities performed by people themselves in the case of very severe limitations (e.g. JACO) [15, 16]. However, many of these simple assistive tools are only usable for a specific task, while the fully robotic systems allow more functionality, but are not portable (except when mounted on a wheelchair), very expensive and too bulky to use unobtrusively in daily life. Furthermore, these fully robotic systems often completely substitute the function of the person [17-19]. Therefore, new technological innovations are needed that can support the arms and hands directly in a wide range of functional tasks. A promising approach is a wearable soft robotic device assisting a person's own function.

Another approach to improve hand motor function is hand exercise training consisting of high repetition and intensive task-specific exercises [20, 21]. However, intensive conventional hand training exercise takes a lot of effort from patients and therapists and is expensive [22, 23]. Therefore, development of robotic devices that can assist in providing training of upper limb motor function in people with functional limitations and disabilities is increasing rapidly [13, 24]. First, these robotic devices can increase independent training with more intensive and repetitive exercises [20]. Second, robotic devices can give adequate objective and reliable

feedback about patients' progress and performance during therapy [25]. Finally, research shows that robot-assisted therapy can reduce motor impairments of the upper limb in stroke patients [24-26].

When as assistive robotic device is wearable and usable in daily life, the opportunity exists to combine direct assistance from a robotic device with exercise training. With a wearable assistive device, performance of functional activities can be enhanced directly, while using the arms and hands repeatedly during functional daily activities, which provides intensive and task-specific training at the same time. Ultimately, it is possible that unsupported arm and hand function improves after prolonged use. To allow prolonged use of such an robotic device in everyday activities at home, an easy to use and affordable system (ironHand (iH) system) is developed within the current project, to support elderly people and patients with hand motor problems during ADL and rehabilitation, based on the concept of a wearable soft-robotic glove. The iH system provides support for grip and hand opening in a natural and intuitive way, but only if the user initiates the movement actively. Furthermore, it will give only the amount of support that is needed. This will make sure that people maintain an active contribution to movements at all time. By adding a personalized computer gaming environment, specific training exercises can be provided as well. Therefore, this study explores whether prolonged use of such an assisting glove during ADL at home can enhance functional performance, and compare this with applying such a robotic device as a training tool at home. By comparing the assistive function with the training function, it is assessed whether assistance or training contributes most to the potential effects.

In the first stage of user testing with the iH system during 2015 (P15-11), both user acceptance and impact on functional performance were investigated [27]. Participants perceived the concept of the iH system to be useful, pleasant and meaningful. Although no major usability issues were observed, functional task performance was slower with assistance from the iH system than without iH system. A continuation of the learning curve during functional task performance after 3 repetitions across 2 sessions indicates that increased performance can be expected in case of a longer time to get acquainted to the iH system. Therefore, design adaptations of the iH system were done to further improve system performance and usability. There were no adverse events observed or reported during or after the study. In the current second stage of user testing, the adapted iH system will be tested in-depth and in a larger group of users. In contrast to the focus on user acceptance in the previous testing phase, the focus of the current study is on the therapeutic effects of the iH system (relating to the assisting and training function, respectively) on functional performance, in addition to user acceptance, actual use and potential impact on quality of life (QoL).

2. OBJECTIVES

Primary Objective:

- To examine whether prolonged use of the iH system for assisting and/or training at home improves functional performance of the most-affected arm and hand

Secondary Objective(s):

- To explore the user acceptance of using the iH system at home, either as assistive or as training tool, including usability, satisfaction, motivation and compliance
- To examine changes in hand strength due to prolonged use of the iH system (either as assistive or as training tool)
- To examine whether elderly or patients improve their overall QoL after prolonged use of the iH system (either as assistance or as training tool)
- To examine changes in amount of use of the most-affected hand after using the iH system as assistance or as training tool
- To explore potential relations between user acceptance, amount of use, changes in hand function and QoL

3. STUDY DESIGN

The present study examines the therapeutic effect of the iH system, either used as assistance or as training tool at home, to improve unsupported arm and hand function of both elderly and patients with hand function problems in ADL. Since this study is part of a European project, similar tests will be done in Sweden and Switzerland besides the Netherlands. The part described in this protocol represents the Dutch part of the study, which involves the participation of the National Foundation for the Elderly (NFE, Bunnik) and Roessingh Research and Development (RRD, Enschede).

This study consists of a randomized controlled trial with two evaluation measurements. The baseline measurement will be performed a week before the start of the study (T0). Afterwards, participants of both the elderly and patient population (via two separate randomization procedures) will be randomized into three equal groups. Group 1 will use the iH assistive system (AS) during ADL at home (iH assistive group), group 2 will use the iH therapeutic system (TS) as a training tool at home (iH therapeutic group) and group 3 will not perform any intervention at home (control group) during the intervention period of four weeks. Within one week after the intervention period a post-evaluation will be performed (T1). During both evaluation sessions, the participants will perform various hand function tests with and without the glove to assess the therapeutic effect of the iH system (modes), as well as the direct influence of the glove assistance. NFE and RRD have different sizes of both left and right hand gloves available to ensure that the participant has the most suitable glove for him/her. An overview of the study is given in Figure 1.

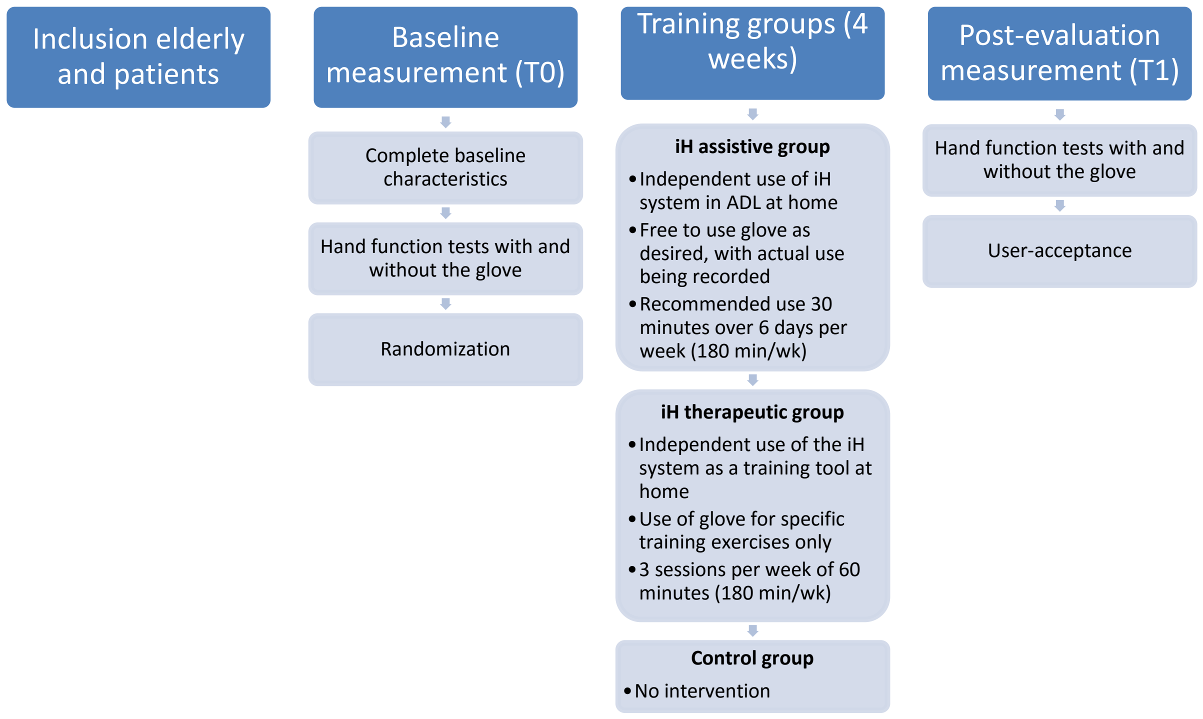

Figure 1. Flowchart study.

4. STUDY POPULATION

4.1 Population (base)

In the present study, NFE and RRD together will recruit 27 elderly participants with perceived hand function problems and 15 patients diagnosed with stroke. The elderly will be selected from the current databases or network of NFE and RRD to participate in this study. The stroke patients will be recruited at Roessingh Center for Rehabilitation, Enschede. A rehabilitation physician will identify stroke patients suitable for the study, based on the selection criteria.
After the selection of suitable subjects, the researcher will contact the potential participants by phone and will provide him/her, both verbally and in writing, with the necessary information about the study, if the person is interested. The information letter explains the goal of the study and their expected involvement. All subjects will be informed that they can stop their involvement at any moment, without giving any reason and without experiencing any disadvantage. All subjects will have to provide written informed consent, prior to their involvement in the study.

4.2 Inclusion criteria

In order to be eligible to participate in this study, the elderly population must meet all of the following criteria:

- Elderly adults over the age of 55 years
- Experience difficulties in performing ADL due to a decline in hand function
- Absence of wounds on their hands that can give a problem when using the glove
- Absence of severe contractures limiting passive range of motion
- Absence of co-morbidities limiting functional use/performance of the arms/hands
- People should have at least 10 degrees of active flexion and extension of the PIP
- Sufficient cognitive status to understand two-step instructions
- Having (corrected to) normal vision
- Living at home
- Provided written informed consent

In order to be eligible to participate in this study, the stroke population must meet all of the following criteria:

- Patients with hand function problems who are clinically diagnosed by a physician with stroke (unilateral ischemic or hemorrhagic stroke)
- Patients over the age of 55 years
- Time since onset of disease is at least 6 months

- Discharged from specific arm/hand therapy
- Absence of severe spasticity of the hand (≤2 points on Ashworth Scale)
- Absence of wounds on their hands that can give a problem when using the glove
- Absence of severe contractures limiting passive range of motion
- Absence of co-morbidities limiting functional use/performance of the arms/hands
- People should have at least 10 degrees of active flexion and extension of the PIP
- Sufficient cognitive status to understand two-step instructions
- Having (corrected to) normal vision
- Living at home
- Provided written informed consent

4.3 Exclusion criteria

A potential subject of either study population who meets any of the following criteria will be excluded from participation in the study in the case of:

- Severe sensory problems of the most-affected hand
- Severe acute pain of the most-affected hand
- Participation in other studies that can affect functional performance of the arm and hand
- Insufficient knowledge of the Dutch language to understand the purpose or methods of the study

4.4 Sample size calculation

The nature of the current study is explorative, to gain understanding about the potential of a wearable robotic glove to support functional abilities and/or improve arm/hand function, in addition to examining users' perspectives of using such technology to support their ability to perform ADL. Therefore, a power calculation is not applicable. The numbers are based on what is believed to be sufficient to gather the intended information and will be feasible and practical to achieve during the course of the study. The study will be replicated with the same intended sample sizes of elderly and stroke patients in Sweden and Switzerland. For the combined study, we are aiming at collecting data from 25 elderly per group and 15 stroke patients per group, which are common, sufficiently powered, sample sizes in other rehabilitation interventions for the upper extremity. So, at least 9 elderly (x3 = 27) and 5 stroke patients (x3 = 15) should be recruited per group in each of the 3 countries.

5. TREATMENT OF SUBJECTS

The researchers involved in the study receive an extensive training about how to handle and operate the iH system by personnel from the technical project partners prior to the start of the study. Also, they are instructed on how to explain use of the iH system to participants, following a standard procedure.

Two evaluation sessions will be performed. The baseline assessment will take place one week before the intervention period and the post-evaluation assessment will take place within one week after the intervention period. During both evaluations first a calibration measurement will be performed, to assess the proper amount of support to be set in strength and hand opening. Thereafter, an extensive set of hand function tests will be performed. These hand function tests will be performed with and without the glove to assess the direct impact of the iH system. At the end of the hand function tests, participants will be asked about their experiences and perceived ability of using the iH system (either as assistance or training tool).

5.1 Investigational product/treatment

After the baseline assessment, the participants will be randomly assigned to one of the three experimental groups.

1. iH assistive group

The iH assistive group will use the wearable robotic glove during ADL at home for 4 weeks. The participants are free to choose for which activities, when and for how long they use the iH AS. However, it is recommended to use the iH AS at least 180 minutes a week during the most common ADL such as dressing/undressing, eating/drinking, functional transfers and personal hygiene. The iH AS will automatically register the amount of use of the device and the participants will register the amount of use in a diary. During the baseline assessment at RRD, the participants will perform a calibration measurement to assess the proper amount of support to be set in strength and hand opening. At the end of this initial session, instructions about all aspects of iH AS use will be given, demonstrated to and practiced with the participant, until the researchers are confident that the participant knows how to use the system at home properly. The instructions will be repeated upon installation of the iH system at their home.

During the intervention period, researchers will contact the participants once every week by phone, e-mail or a visit, to make sure the participant is doing well and answering any questions about their arm/hand function and ADL or responding to potential problems that might arise. In addition, there will be a phone number available the participants can call in case of problems. The participants will also be provided with a manual with most important information about the system.

<u>2. iH therapeutic group</u>

The iH therapeutic group will use the wearable robotic glove as a training tool at home for 4 weeks to train their hand function. These participants will be recommended to use the iH TS for (a minimum of) 180 minutes a week. At the end of the baseline session, instructions about all aspects of iH TS use will be given, demonstrated to and practiced with the participant, until the researchers are confident that the participant knows how to use the system at home properly. The instructions will be repeated upon installation of the iH system at their home.

During training, the iH TS will support grip strength and hand opening in a similar way as for the assisting system, but now as applied during training exercises only. These exercises are available on a laptop, running training software with specific exercises for the hand to improve hand motor function and abilities. Based on the sensor readings in the glove, games on a computer screen are controlled by active hand movements of the participant. It is possible to play these games with resistance in the therapeutic glove. However, all exercises are adapted to each person's ability by the therapist.

The participants will follow the instructions on the screen to start each training. The training always starts with a calibration procedure disguised as a game, in order to set the proper game level and the proper amount of support by the device. After the calibration game, the subject will play an exercise game from the therapy plan (is created by the therapist in advance) dependent on the ability and needs of the participant. During the training, the participants will receive feedback to keep him/her motivated. The iH TS will automatically record the actual training time.

Therapeutic exercises

The therapeutic exercises will consist of three different therapeutic goals:

Exercise 1: Submarine

Therapy goal: Simultaneous finger coordination (position)

The exercise will require the user to control a robotic submarine equipped with 5 robotic arms that move according to the user's finger angle (signals are coming from the glove). The user will be required to adapt different hand postures in order to collect coins or avoid undesirable items (e.g. bombs). Different coin and bomb locations will encourage the users to adapt different postures and to train the simultaneous coordination of finger flexion and extension.

Exercise 2: High flyer

Therapy goal: Hand strength

The exercise will require the user to control up and down movements of a character on the screen using hand opening and closing movements. The user will be required to modulate their hand aperture in order to collect points. As the level of difficulty progresses, the glove will provide resistance in either closing or opening the hand (according to the therapeutic need set by the therapist).

Exercise 3: Birds on string
Therapy goal: Sequential finger coordination
The exercise will require the user to repeat a sequence of thumb opposition movements. The sequence of movements will be presented to the user on the computer screen by a combination of visual and auditory cues. As the level of difficulty progresses, the sequence of movements will become more complex and will require the user to make use of more fingers in order to achieve the goal.

During the intervention period, researchers will contact the participants once every week by phone, e-mail or a visit, to make sure the participant is doing well and answering any questions about their arm/hand function and ADL or responding to potential problems that might arise. In addition, there will be a phone number available the participants can call in case of problems. The participants will also be provided with a manual with most important information about the system.

3. <u>Control group</u>
The participants of the control group do not follow a specific intervention during the intervention period. They will continue with their normal activity pattern of their most-affected hand. To keep the personal attention the same in comparison with the experimental groups, also these participants will be contacted and asked about their arm/hand function and ADL once every week by phone, e-mail or a visit by the researchers. In addition, there will be a phone number available the participants can call in case of problems.

5.2 Use of co-intervention (if applicable)
Use of co-intervention is not applicable.

5.3 Escape medication (if applicable)
Escape medication is not applicable.

6. INVESTIGATIONAL PRODUCT

6.1 Name and description of investigational product(s)

The wearable robotic glove (iH system) is based on an existing grip-enhancing glove, SEM glove . The iH system is specifically designed towards the needs of elderly people with declining hand function. The iH system falls under Rule 9 of the Medical device directive, and is considered to be a class IIa device.

The iH system is based on the concept of a soft-robotic glove. The main characteristics of the system are:

- A cable-driven glove that can provide assistive force to open and close the hand during everyday activities or therapeutic exercises;
- The assistive force is triggered by an “intention detection” logic that reacts to movement initiation by the user;
- The glove can be connected to an external PC that allows the user to perform specific, computer-game-like exercises tied to functional tasks in order to keep the motivation to remain active.

The iH system is composed of two main parts: an iH AS and an iH TS (see Figure 2). The iH AS consists of a (1) Control unit and a (2) Glove. The iH TS consists of a (3) Therapeutic platform and (4) Therapeutic software.

The iH AS can be used by itself, i.e. without the iH TS. In this configuration*,* the iH system improves *i)* the hand grip strength and endurance following the users grip intention, *ii)* the hand opening functionality by using passive leaf spring, and *iii)* the agility of the fingers. It can be used when additional grip strength and endurance is desired, for training on everyday activities and to monitor the user’s performance.

The iH AS can also be used in combination to the iH TS to provide specific hand movement therapy and assessments. In this configuration, the iH system supports specific exercises in order to increase muscle strength, coordination, fine motor skills and range of motion in different joints with the aim to improve or maintain motor function. The sensors integrated in the glove allow for control of movement-dependent games.

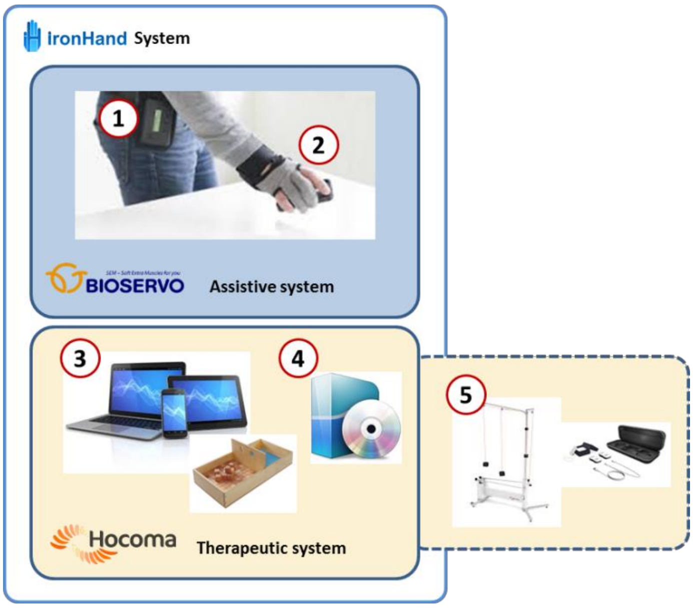

Figure 2. ironHand system

The iH system consists of the following parts (Figure 2):

1) Control unit - The control unit contains a battery for power source, one motor for each finger that receives extra force and a microcontroller that controls the iH AS functionality. A cord connects the control unit with the glove and holds the artificial tendons as well as electrical cables for sensors. Close to the glove there is a connection that can be opened, separating the glove from the control unit. This gives the possibility to change between gloves or replace an old glove. An embedded software in the control unit adjusts the amount of assistive force to help the user open or close the hand. When an object is held with the aid of the iH AS, the touch sensors on the finger tips signal to the control unit which pulls the tendons such that the force in the grip becomes larger. The extra force applied by the glove is in proportion to the force applied by the user. Hence, the user can control (increase or decrease) the extra force applied by the iH AS.

2) Glove - The main purpose of the glove is to apply the forces generated by the motors in the control unit and to provide the control unit with sensory input from touch sensors at the fingertips. The forces are applied by artificial tendons that are sewn into the glove along the length of the fingers. The glove works together with the control unit that is placed on the hip or on the back of the user. The glove has a slim design and the same look and feel as a regular glove; therefore, it can be worn as any other glove.

The glove functionality also consists of a hand opening function realized by the use of passive (leaf) springs attached to the top of the hand. The springs cover the fingers and the back of

the hand. This functionality means that the glove should be capable of actively controlling forces for flexion and extension of the fingers.

3) Therapeutic platform - The therapeutic platform refers to a computing system (e.g. PC, laptop or tablet) to which the iH AS is connected. This allows the user to train with motivating game-like exercises and visualize his progress through automated reports.

4) Therapeutic software – Additional therapeutic functionality of the iH system is embedded in a therapeutic software. Software development complies with IEC62304:2006 and IEC 60601-1:2005 standards. This software includes the following functionalities: assessments, connectivity, database, exercises, software architecture, safety mechanisms and user interface.

The following configurations will be used during the research studies described in this protocol:

Configuration I - Orthotic mode

The system is considered to operate in *orthotic mode* when the iH AS is not connected to the iH TS. This configuration will be used by the iH assistive group at home. During both evaluation sessions, participants will perform several hand function tests with and without the glove in orthotic mode, wearing it on their most-affected hand to support their hand as desired and needed. The direct impact of the iH AS will be explored, but also user acceptance will be explored.

Configuration II - Therapeutic mode

The system is considered to operate in *therapeutic mode* when the iH AS is connected to the iH TS. In this configuration, the user can benefit from additional therapeutic training in a motivating environment. This configuration will be used by the iH therapeutic group at home and will not be used in both evaluation sessions.

6.2 Summary of findings from non-clinical studies

Findings from non-clinical studies are not applicable in the case of this biomedical device, which is developed for specific interaction with the human body. See IMDD for detailed information about technical testing and product (component) functionality tests.

6.3 Summary of findings from clinical studies

Elderly, stroke patients and healthcare professionals, participating in focus groups, have specified requirements regarding: 1) activities that need support of assistive technology, 2)

design of wearable robotic devices for hand support, and 3) application of assistive technology as training tool at home. Assistive technology for the support of the hand is considered valuable by users for assisting ADL, but only if the device is wearable, compact, lightweight, easy to use, quickly initialized, washable and only supports the particular function(s) that an individual need(s) assistance with, without taking over existing function(s) from the user [28]. The second prototype of the iH system is already tested on feasibility, in terms of user acceptance and impact on functional task performance. Overall, participants perceived the concept of the iH system to be useful, pleasant and meaningful. The learning curve in functional performance is promising for increased performance in case of a longer time to get acquainted to the iH system. However, design adaptations of the iH system are needed to further improve performance with the glove [Article submitted to conference ICT4ageingwell] . In this next stage of user testing, the adapted iH system will be tested in a larger group of users regarding the therapeutic effect of the iH system. Several studies suggest that this approach may provide therapeutic benefits to the user (see *Literature review references* below). Furthermore, the feasibility study of Nilsson et al. 2013 about the SEM-glove showed already that such a system can be beneficial for participants with impaired hand function. In addition, the clinical evaluation report of the predicate device SEM™ Glove is considered to provide partial clinical evidence for the iH System when used in *orthotic mode* (see IMDD for more information) . The rehabilitation effects of the use in *orthotic mode* are not covered by the references document, but need further clinical evaluation as described above.

Literature review references

Barreca S, Wolf SL, Fasoli S, Bohannon R. Treatment interventions for the paretic upper limb of stroke survivors: a critical review. Neurorehabil Neural Repair. 2003;17:220-6.

Batavia AI, Hammer GS. Toward the development of consumer-based criteria for the evaluation of assistive devices. J Rehabil Res Dev 1990;27:425-36.

Brooks NA. Users responses to assistive devices for physical disability. Social Science and Medicine 1991;32(12);1417-24.

Buma F, Kwakkel G, Ramsey N. Understanding upper limb recovery after stroke. Restorative Neurology and Neuroscience 2013;31(6):707-22.

Colombo R, Pisano F, Mazzone A, Delconte C, Micera S, Carrozza MC, Dario P, Minuco G. Design strategies to improve patient motivation during robot-aided rehabilitation. Journal of NeuroEngineering and Rehabilitation 2007;4:3.

Demain S, Burridge J, Ellis-Hill C, Hughes A, Yardley L, Tedesco-Triccas L, Swain I. Assistive technologies after stroke: self-management or fending for yourself? A focus group study. BMC Health Services Research 2013;13:334.

Frappier J. Clinico-economic study of the JACO robotic arm for powered wheelchair users with upper-extremity disabilities, by Data4Actions Inc. for Kinova, Canada 2011.

Hidler J, Nichols D, Pelliccio M, Brady K. Advances in the understanding and treatment of stroke impairment using robotic devices. Top Stroke Rehabil 2005;42:239-52.

Hochstenbach-Waelen A, Seelen HAM. Embracing change: practical and theoretical considerations for successful implementation of technology assisting upper limb training in stroke. Journal of NeuroEngineering and Rehabilitation 2012;9:52

Huang LL, Lee CF, Hsieh CL, Chen MH. Upper extremity rehabilitation equipment for stroke patients in Taiwan: usage problems and improvement needs. Occupational Therapy International 2013;20:205-14.

Kennedy AP, Rogers AE, Bower P. Support for self-care for patients with chronic disease. BMJ 2007;335:968-70.

Kwakkel G. Impact of intensity of practice after stroke: issues for consideration. Disabil Rehabil. 2006;28(13-14):823-30.

Latham NK, Jette DU, Coster W, Richards L, Smout RJ, James RA, Gassaway J, Horn SD. Occupational therapy activities and intervention techniques for clients with stroke in six rehabilitation hospitals. The American Journal of Occupational Therapy 2006;60(4):369-378.

Lee M, Rittenhouse M, Hussein AA. Design issues for therapeutic robot systems: results from a survey of physiotherapists. J Intell Robot Syst 2005;42:239-52.

Lu EC, Wang RH, Hebert D, Boger J, Galea MP, Mihailidis A. The development of an upper limb stroke rehabilitation robot: identification of clinical practices and design requirements through a survey of therapists. Disability and Rehabilitation: Assistive Technology 2011;6(5):420-31.

Meng Q, Lee MH. Design issues for assistive robotics for the elderly. Advanced Engineering Informatics 2006;20:171-86.

Römer GRBE, Stuyt HJA, Peters A. Cost-savings and economic benefits due to the Assistive Robotic Manipulator (ARM), presented at the International Conference on Rehabilitation Robotics (ICORR) 2005, Chicago (IL), USA.

Saposnik G, Teasell R, Mamdani M, Hall J, McIlroy W, Cheung D, Thorpe KE, Cohen LG, Bayley M. Effectiveness of virtual reality using Wii gaming technology in stroke rehabilitation: a pilot randomized clinical trial and proof of principle. Stroke 2010;41:1477-1484.

Schaechter JD. Motor rehabilitation and brain plasticity after hemiparetic stroke. Prog Neurobiol. 2004;73:61-72.

Shakya Y, Johnson M. A mobile robot therapist for under-supervised training with robot/computer assisted motivating systems. 30th Annual International IEEE EMBC Conference 2008; Vancouver (Canada); August 20-24.

Stehle C, Albrecht-Buehler A. Developing more desirable products for stroke survivors. Top Stroke Rehabilitation 2008;15(2):109-17.

Sugar TG, He J, Koeneman EJ, Koeneman JB, Herman R, Huang H, Schultz RS, Herring DE, Wanberg J, Balasubramanian S, Swenson P, Ward JA. Design and control of RUPERT: a device for robotic upper extremity repetitive therapy. IEEE Transactions on neural systems and rehabilitation engineering 2007;15(3):336-46.

Timmermans AA, Seelen HA, Willmann RD, Kingma H. Technology-assisted training of arm-hand skills in stroke: concepts on reacquisition of motor control and therapist guidelines for rehabilitation technology design. Journal of NeuroEngineering Rehabilitation 2009;6:1.

Van der Heide LA, van Ninhuijs B, Bergsma A, Gelderblom GJ, van der Pijl DJ, de Witte LP. An overview and categorizatioin of dynamic arm supports for people with decreased arm function. Prosthet Orthot Int 2013 Aug 15.

Wardlaw JM, Kournellis P, Liu M. Thrombolysis (different doses, routes of administration and agents) for acute stroke. Cochrane Database of Systematic Reviews 2009;5.

6.4 Summary of known and potential risks and benefits

The iH system may have a beneficial effect on hand function, by directly improving functional task performance or by using it as a training tool. It might be possible that the functional use of the hand improves, allowing people to be more active in ADL and to maintain or improve their health status. However, the exact benefit cannot be predicted, because this is the topic of the current research.

The risks for the subjects are limited to a minimum. The iH system is a device that facilitates hand grip and opening as initiated by the user him/herself. It provides support only when necessary based on voluntary, active initiation by the person him/herself. Furthermore, the iH system is a so-called soft-robotics device, constructed from soft materials that are comfortable to wear and compliant with human movement. This prevents potential occurrence of pressure points for example. All movements conducted during the study will consist of hand movements that normally occur in ADL and are within the abilities of each individual. Additionally, all the evaluation measurements used in these studies are non-invasive and involve no risks for the subjects.

In the first stage of user testing with the iH system during 2015 (P15-11), no adverse events were observed. Elderly noticed the support of the iH system and considered the support as pleasant and meaningful. However, several design changes (e.g. fabric of the glove to improve sensation) based on the results of the feasibility study are necessary to further enhance the chance for uptake of the iH system in daily life. This should also improve functional task performance with the glove beyond performance without the glove, before actual use of such assistive system will be beneficial for (and adopted by) elderly [27].

For further information, see IMDD and the associated risk management reports.

6.5 Description and justification of route of administration and dosage

The glove applies force to the hand following the same biomechanical constraints as the corresponding muscles. This means that there is no risk that the glove causes unnatural movement of the hand and/or fingers (such as hyperextension). The force to be applied as well as sensitivity of the system will be adjustable to suit the user’s needs and limitations by trained personnel. The user will not be able to do any modifications to the configuration of the iH system. It is only possible to choose one of the three different modes with different sensitivity and force values to optimize it for each circumstance. The maximum force of the system is mechanically limited by the motors to a level that is comparable to an average female adult, which means that harmful levels of pressure and/or power can never be reached.

In addition, it is important to remember that the device follows the user’s own intention. This means that if the user grasps an object harder the glove will provide more force (up to the limited maximum level) but as soon as the user initiates to release the grasp the force applied by the glove will decrease. This gives good feedback to the user and lowers the risk for harm caused by the user dropping things (coffee cup, hot pot, knife etc.).

The same technology has been successfully used in the CE marked SEM™ Glove for a number of years without leading to incidents and/or causing harm. Furthermore, the feasibility study of Nilsson et al. 2013 [29] showed the potential benefit for participants with impaired hand function when they will use such a device as the SEM-glove. Participants with impaired hand function improved their grip while using the SEM-glove.

6.6 Dosages, dosage modifications and method of administration

Not applicable.

6.7 Preparation and labelling of Investigational Medicinal Product

Not applicable.

6.8 Drug accountability

Not applicable.

7. NON-INVESTIGATIONAL PRODUCT

Not applicable.

8. METHODS

8.1 Study parameters/endpoints

8.1.1 Main study parameter/endpoint

- Jebsen-Taylor hand function test (JTHFT)

8.1.2 Secondary study parameters/endpoints (if applicable)

The following parameters will be measured during the baseline and post-evaluation assessment (T0 and T1) to obtain more insight in changes associated with dexterity on impairment, activity and participation levels:

- Maximal handgrip strength (Jamar dynamometer)
- Handgrip endurance
- Pinch grip strength
- Box and Block test
- Motor activity Log (MAL)
- SF-36 questionnaire

In addition, the following secondary parameters about user acceptance will be measured at the end of the study (T1):

- System Usability Scale (SUS) questionnaire
- Intrinsic Motivation Inventory (IMI) questionnaire
- Semi-structured interview about user's experience

8.1.3 Other study parameters (if applicable)

In addition to the parameters measured during the evaluation measurements, during the course of the study the actual amount of use of the glove and/or exercises is logged by the iH system or registered by the participant.

Other parameters to be collected are descriptive subject characteristics (Appendix 1), which will be documented at the baseline measurement:

- Name
- Date of birth
- Sex
- Height
- Weight
- Most-affected side
- Dominant side before and after impairment
- Date of diagnosis and impairment/pathology (only for patient population)

- Location of the stroke (size based on imaging (e.g. CT-scan), if it is present in the subjects status; only for stroke population)

8.2 Randomisation, blinding and treatment allocation

The patient population will be stratified by diagnosis. Then participants will be randomly assigned via a list of random numbers (randomisation list) to one of the three intervention groups to provide an equal number of participants in all groups. (block randomisation). The randomization procedure will take place after the baseline measurement.

In addition, the order of the functional tests with and without wearing the glove will be randomized within one session before the start of the functional hand function tests in both evaluation sessions. The numbers 1-10 will be distributed over 10 envelopes. For each participant, the researcher will take one envelope from these 10 envelopes to randomize the order of functional tests with and without the glove. If the researcher draws an even number, the participant will perform all functional tasks with the glove first during both evaluations. If the researcher draws an odd number, the participant will perform all functional tasks without the glove first during both evaluations.

The study cannot be blinded for the participants or researchers.

8.3 Study procedures

Each participant will have a baseline (T0) and post-training evaluation session (T1). The baseline measurement will consist of a short introduction and instruction about how to use the iH system, followed by several hand function tests with and without the use of the glove to assess the direct influence of the iH system before prolonged use. The post-training measurement follows the same procedure as the baseline measurement, with addition of assessment of user acceptance through several questionnaires. The complete list of procedures of the study is shown in Table 1 and 2.

Table 1: Study protocol for baseline measurement

	Activity	Time (min.)
1	Introduction about the study	10
2	Complete subject characteristics	5
3	Explanation of the glove	10
4	Trying out the glove	15
5	Maximal handgrip strength	15
6	Handgrip endurance	5
7	Pinch grip strength	10
8	Jebsen-Taylor Hand Function Test (JTHFT)	25
9	Box and Block Test (BBT)	10
10	Motor Activity Log (MAL)	20
11	SF-36 questionnaire	10
12	Instructions for home use	15
	**Total:**	**150 min**

Table 2: Study protocol for post-evaluation measurement

	Activity	Time (min.)
1	Short evaluation about the intervention period	10
2	Short trying out the glove	5
3	Maximal handgrip strength	10
4	Handgrip endurance	15
5	Pinch grip strength	10
6	Jebsen-Taylor Hand Function Test (JTHFT)	25
7	Box and Block Test (BBT)	10
8	Motor Activity Log (MAL)	20
9	SF-36 questionnaire	10
10	System Usability Scale (SUS)	5
11	Intrinsic Motivation Inventory (IMI)	15
12	Semi-structured interview	15
	**Total:**	**150 min**

8.3.1 Evaluation measurements – Main study parameters

Jebsen-Taylor hand function test (JTHFT) (Appendix 2)

The JTHFT is a reliable and valid test to evaluate functional hand motor function in different patient groups and healthy people of various ages [30]. The test consists of 7 different unilateral hand skill tasks related to ADL: (1) writing a sentence of 24 characters (2) turning over 7.6- x 12.7-cm cards (3) picking up small, common objects (e.g., paper clips, coins and bottle caps) and move these to a box (4) stacking checkers (test of eye-hand coordination) (5) simulated feeding (e.g. teaspoon with beans) (6) picking up large empty cans (7) moving weighted (450 g) cans. The time of each different task will be recorded in seconds and the different times summed is the total score for the test [30, 31].

8.3.2 Evaluation measurements – Secondary study parameters

Maximal handgrip strength

The maximal handgrip strength will be measured with a Jamar dynamometer [32, 33]. Each participant will sit comfortably with the elbow of the most-affected arm close to their body, flexed 90 degrees, holding the dynamometer in the most-affected hand. The other parts of the body are not allowed to move or help to give more strength. The participant will squeeze the handgrip of the dynamometer maximally, which is maintained for 5 seconds. There will be three attempts and between the different attempts is at least 60 seconds rest. The best of the three attempts will count. This measurement will be done by the less-affected hand as well for reference purposes.

Handgrip endurance

Handgrip endurance will be measured in a static situation. In the static situation, the participants will squeeze the Jamar dynamometer maximally for 30 seconds. The formula for analysing fatigue in a static situation is last second / first second, to measure the change in power (N) in percentage [34]. The participant has one attempt for both conditions with and without the glove.

Maximal pinch strength

The maximal pinch strength will be assessed with a Jamar pinch Gauge dynamometer. The pinch strength will be measured between the index finger and the thumb and the ring finger and the thumb. The subject will be seated comfortably with the elbow close to their body, flexed 90 degrees and resting on a table. The subject will in all attempts grasp the pinch dynamometer with the distal segment and ventral side of the thumb and finger. In all attempts, the subject will squeeze in the pinch dynamometer maximally with the two fingers for at least 5 seconds. The other fingers are not allowed to give any support. The subject will get 3 attempts for each test and the best one counts. Between all the attempts is at least 60 seconds rest.

Box and Blocks test (BBT) (Appendix 3)

The BBT is a simple, reliable and valid measurement to measure manual dexterity of elderly people [35]. In the test, the participant grasps blocks with his or her most-affected hand and transfers these from one box to the other adjacent box. The maximum time for this measurement is one minute and the participant has to transfer as many blocks as possible, one at a time. At the end of the measurement, the number of transferred blocks is counted. More blocks transported indicate a better gross manual dexterity [35, 36].

Motor Activity Log (MAL) (Appendix 4)

The MAL is a semi-structured questionnaire to explore the self-perceived amount of use and quality of movement of the affected arm and hand in stroke patients in ADL. This questionnaire consists of 26 activities and has excellent test- retest reliability for both scores of each activity [37]. Each activity will be scored by the participant for quality of movement and amount of use of the upper extremity. The possible scores for both aspects can range from 0 to 5 [37, 38].

SF-36 questionnaire (Appendix 5)

The SF-36 is a 36 item questionnaire to assess health perception of people. It is a validated and reliable assessment for measuring health perception [39]. The questionnaire consists of multi- item dimensions about physical and mental well-being of the participant. The scores of the questionnaire will be converted to a 0 – 100 scale, where a higher score indicates a better QoL [39, 40].

System Usability Scale (SUS) (Appendix 6)

The SUS is a simple, valid and reliable questionnaire for systems' usability. It uses a 5- point Likert scale for 10 questions about ease of use and related issues. The answers can range from 'strongly disagree' till 'strongly agree'. The total score of the questions will be multiplied by 2.5, so that the maximum score is 100 [41].

Intrinsic Motivation Inventory (IMI) (Appendix 7)

The IMI questionnaire is a simple, easy to use, valid and reliable questionnaire to assess individuals' intrinsic motivation during any specific exercise activity [42-44]. The items of the IMI questionnaire will be scored by the participant on a 7-point Likert scale in the range from 'not at all true' till 'very true' [42].

8.4 Withdrawal of individual subjects

Subjects can leave the study at any time for any reason if they wish to do so without any consequences. The investigator can decide to withdraw a subject from the study for urgent medical reasons.

8.4.1 Specific criteria for withdrawal (if applicable)

A subject will be withdrawn if the subjects' health is affected or other adverse effects are encountered during the use of the device.

8.5 Replacement of individual subjects after withdrawal

If possible within the duration of the study, the subject will be replaced after withdrawal.

8.6 Follow-up of subjects withdrawn from treatment

There will be no follow-up for the subjects after withdrawal from the study.

8.7 Premature termination of the study

The study will be terminated prematurely if serious adverse events occur during the study procedures. In such situations, the subjects will be informed as quickly as possible by the accredited METC and laboratory manager.

9. SAFETY REPORTING

9.1 Temporary halt for reasons of subject safety

In accordance to section 10, subsection 4, of the WMO, the sponsor will suspend the study if there is sufficient ground that continuation of the study will jeopardise subject health or safety. The sponsor will notify the accredited METC without undue delay of a temporary halt including the reason for such an action. The study will be suspended pending a further positive decision by the accredited METC. The investigator will take care that all subjects are kept informed.

9.2 AEs, SAEs and SUSARs

9.2.1 Adverse events (AEs)

Adverse events are defined as any undesirable experience occurring to a subject during the study, whether or not considered related to the use of the iH system. All adverse events reported spontaneously by the subject or observed by the investigator or his staff will be recorded.

9.2.2 Serious adverse events (SAEs)

A serious adverse event is any untoward medical occurrence or effect that

- results in death;
- is life threatening (at the time of the event);
- requires hospitalisation or prolongation of existing inpatients' hospitalisation;
- results in persistent or significant disability or incapacity;
- is a congenital anomaly or birth defect; or
- any other important medical event that did not result in any of the outcomes listed above due to medical or surgical intervention but could have been based upon appropriate judgement by the investigator.

An elective hospital admission will not be considered as a serious adverse event.

The sponsor will report the SAEs through the web portal *ToetsingOnline* to the accredited METC that approved the protocol, within 7 days of first knowledge for SAEs that result in death or are life threatening followed by a period of maximum of 8 days to complete the initial preliminary report. All other SAEs will be reported within a period of maximum 15 days after the sponsor has first knowledge of the serious adverse events.

9.2.3 Suspected unexpected serious adverse reactions (SUSARs)

This chapter is not applicable because the iH system is not an investigational medicinal product.

9.3 Annual safety report

This chapter is not applicable because the iH system is not an investigational medicinal product.

9.4 Follow-up of adverse events

All AEs will be followed until they have abated, or until a stable situation has been reached. Depending on the event, follow up may require additional tests or medical procedures as indicated, and/or referral to the general physician or a medical specialist. SAEs need to be reported till end of study within the Netherlands, as defined in the protocol.

9.5 [Data Safety Monitoring Board (DSMB) / Safety Committee]

Not applicable.

10. STATISTICAL ANALYSIS

The data of the outcome measures will be analysed using IBM SPSS Statistics version 23.0. All data will be checked for normal distribution by visual inspection of the q-q plot, the box plot, histogram plot and by the Shapiro-Wilks test, prior to the statistical analyses of the various outcome measures. Descriptive statistics will be used for all outcome measures, using mean ± standard deviation (SD) or median and interquartile range (IQR) as applicable. The overall level of significance will be set at $p < 0.05$.

10.1 Primary study parameter(s) and secondary study parameter(s)

A paired sample t-test or its non-parametric equivalent, the Wilcoxon signed rank test, will be executed to establish the direct/orthotic effect of the iH system for the various hand function outcome measures separately (JTHFT, handgrip strength, BBT, MAL).

In order to assess the effect of the intervention and the differences between the three intervention groups, an one-way ANOVA or the Friedman Test (non-parametric test) will be established for the different outcome measures separately. If a significant difference is found for parametric variables, multiple comparisons are performed with a Bonferroni correction. A Wilcoxon signed rank test for multiple comparisons will be performed using an adjusted P-value of 0.017, if a significant difference for non-parametric variables is found.

In addition, correlation analyses will be used to identify correlations between motivation, usability, actual amount of use and outcome measures for functionality of the hands. The correlation analyses will be established with the parametric Pearson's correlation coefficient or the non-parametric Spearman's correlation coefficient.

10.2 Other study parameters

Descriptive statistics will be used to show the mean ± SD or median and interquartile ranges of the relevant subjects' characteristics.

10.3 Interim analysis (if applicable)

Not applicable.

11. ETHICAL CONSIDERATIONS

11.1 Regulation statement

This study will be conducted according to the principles of the Declaration of Helsinki (64th WMA General Assembly, Fortaleza, Brazil, October 2013) and in accordance with the Medical Research Involving Human Subjects Act (WMO, the Netherlands).

11.2 Recruitment and consent

The elderly population will be selected by the researchers of RRD or NFE based on inclusion and exclusion criteria. The patient population will be selected by a rehabilitation physician and/or therapist. Potential candidates will be asked if they are interested in participation. When expressing interest in participation (to the researcher or rehabilitation physician), subjects will be provided with written and verbal information about the study by the researchers. The subject has one week to consider their decision for involvement in the study. If the subject decides to participate in the study, the subject has to sign the attached informed consent indicating voluntary participation in this study and satisfactory information provision about all aspects of the study.

11.3 Objection by minors or incapacitated subjects (if applicable)

Not applicable.

11.4 Benefits and risks assessment, group relatedness

The iH system may have a beneficial effect on hand function, by directly improving functional task performance or by using it as a training tool. It might be possible that the functional use of the hand improves, allowing people to be more active in ADL and to maintain or improve their health status. However, the exact benefit cannot be predicted, because this is the topic of the current research.

The risks for the subjects are limited to a minimum. The iH system is a device that facilitates hand grip and opening as initiated by the user him/herself. It provides support only when necessary based on voluntary, active initiation by the person him/herself. Furthermore, the iH system is a so-called soft-robotics device, constructed from soft materials that are comfortable to wear and compliant with human movement. All movements conducted during the study will consist of hand movements that normally occur in ADL and within the abilities of each individual. Additionally, all the evaluation measurements used in these studies are non-invasive and involve no risks for the subjects.

11.5 Compensation for injury

The sponsor/investigator has a liability insurance which is in accordance with article 7 of the WMO.
The sponsor (also) has an insurance which is in accordance with the legal requirements in the Netherlands (Article 7 WMO). This insurance provides cover for damage to research subjects through injury or death caused by the study.
The insurance applies to the damage that becomes apparent during the study or within 4 years after the end of the study.

11.6 Incentives (if applicable)

Subjects can receive compensation for travel costs.

12. ADMINISTRATIVE ASPECTS, MONITORING AND PUBLICATION

12.1 Handling and storage of data and documents

Data will be collected and processed according to the European Data Protection Act (95/46/EC and 93/42/EWG) and the European Clinical Trials Directive (2001/20/EC) on Good Clinical Practice in the Conduct of Clinical Trials on Medicinal Products for Human Use. Data will be collected and used for research purposes only by written permission of the subject, according to their informed consent. The data of the relevant subjects' characteristics, questionnaires, clinical trials and laboratory test will be numerically coded. The information that is concealed by the numeric code is only accessible by the investigators. If necessary, the subject can be linked to the data by a subject identification code list which is safeguarded by the investigators. All data will be stored in coded form for the next 15 years. When data is transferred between the project partners, this will concern coded data only. Furthermore, sensitive data will be protected with an encryption. The researchers can use the data for research purposes, such as presentations in a scientific context and conferences without the names and identities of the subjects.

12.2 Monitoring and Quality Assurance

Not applicable.

12.3 Amendments

Amendments are changes made to the research after a favourable opinion by the accredited METC has been given. All amendments will be notified to the METC that gave a favourable opinion.

Non-substantial amendments will not be notified to the accredited METC and the competent authority (CA), but will be recorded and filed by the sponsor. Examples of non-substantial amendments are typing errors and administrative changes like changes in names, telephone numbers and other contact details of involved persons mentioned in the submitted study documentation.

12.4 Annual progress report

The sponsor/investigator will submit a summary of the progress of the trial to the accredited METC once a year. Information will be provided on the date of inclusion of the first subject, numbers of subjects included and numbers of subjects that have completed

the trial, serious adverse events/ serious adverse reactions, other problems, and amendments.

12.5 Temporary halt and (prematurely) end of study report

The investigator/sponsor will notify the accredited METC of the end of the study within a period of 8 weeks. The end of the study is defined as the last patient's last visit.
The sponsor will notify the METC immediately of a temporary halt of the study, including the reason of such an action.
In case the study is ended prematurely, the sponsor will notify the accredited METC within 15 days, including the reasons for the premature termination.
Within one year after the end of the study, the investigator/sponsor will submit a final study report with the results of the study, including any publications/abstracts of the study, to the accredited METC.

12.6 Public disclosure and publication policy

There are no restrictions between sponsor and investigators concerning public disclosure and publication of the research data.

13. STRUCTURED RISK ANALYSIS

Potential risks of the iH system prototype to be used in these research studies were identified and evaluated as described in Figure 3. Risk assessment of the iH system operating in in both orthotic mode and therapeutic mode are covered in the IMDD and its appendices in detail (see Figure 3).

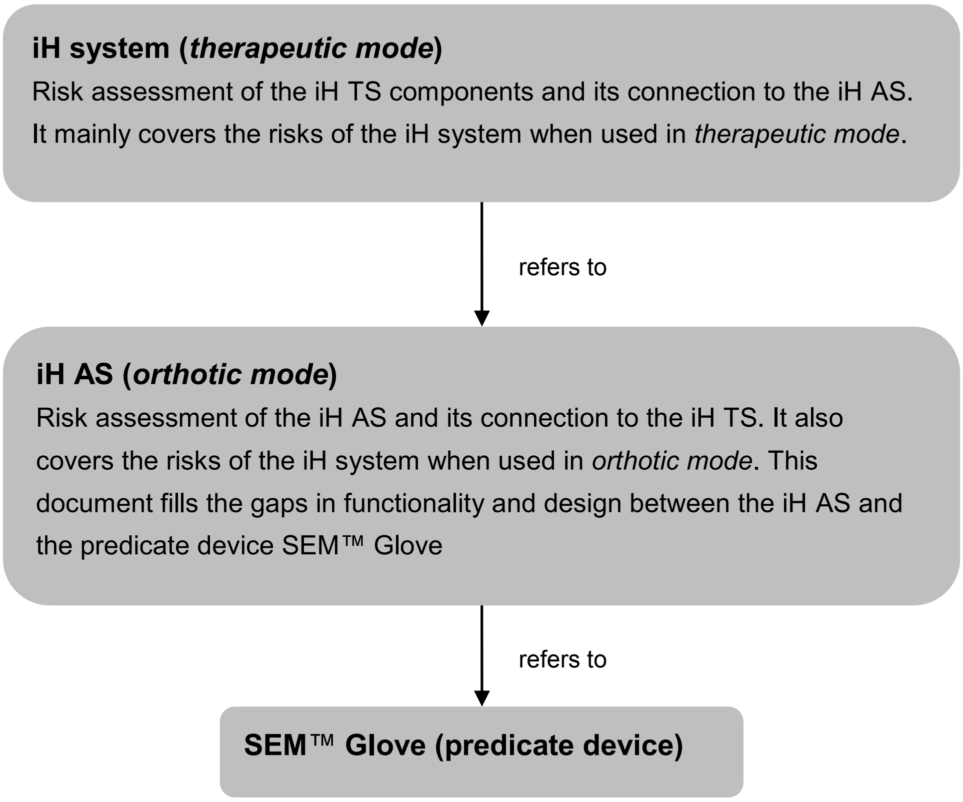

Figure 3. Overview of the risk management documents for the iH system

Risk causes and risk control measures have been reviewed and evaluated in the IMDD and associated documents. The conclusion is, given the intended use of the device, that the overall residual risk is acceptable. There were no identified unacceptable residual risks that may occur in connection with and application of the iH system prototype in this proposed research study.

Risk management will continue during the development and testing of the iH system prototype. Any feedback from usage of the device during the usability test will be immediately considered in the risk management process.

14. REFERENCES

1. Rantanen, T., et al., *Grip strength changes over 27 yr in Japanese-American men.* Journal of Applied Physiology, 1998. **85**(6): p. 2047-2053.
2. Hunter, S. and P. Crome, *Hand function and stroke.* Reviews in Clinical Gerontology, 2002. **12**(01): p. 68-81.
3. Carmeli, E., H. Patish, and R. Coleman, *The Aging Hand.* The Journals of Gerontology Series A: Biological Sciences and Medical Sciences, 2003. **58A**(2): p. 146-152.
4. Kinoshita, H. and P.R. Francis, *A comparison of prehension force control in young and elderly individuals.* European journal of applied physiology and occupational physiology, 1996. **74**(5): p. 450-460.
5. Desrosiers, J., et al., *Age-related changes in upper extremity performance of elderly people: A longitudinal study.* Experimental Gerontology, 1999. **34**: p. 393-405.
6. Langhorne, P., J. Bernhardt, and G. Kwakkel, *Stroke rehabilitation.* The Lancet, 2011. **377**(9778): p. 1693-1702.
7. Covinsky, K.E., et al., *Effect of Arthritis in Middle Age on Older-Age Functioning.* Journal of the American geriatrics society, 2008. **56**(1): p. 23-28.
8. Ranganathan, V.K., et al., *Effects of Aging on Hand Function.* Journal of the American Geriatrics Society, 2001. **49**(11): p. 1478–1484.
9. Rizzoli, R., et al., *Quality of life in sarcopenia and frailty.* Calcified tissue international, 2013. **93**(2): p. 101-120.
10. Mayo, N.E., et al., *Activity, participation, and quality of life 6 months poststroke.* Archives of physical medicine and rehabilitation, 2002. **83**(8): p. 1035-1042.
11. Dominick, K.L., et al., *Health-related quality of life among older adults with arthritis.* Health and Quality of life Outcomes, 2004. **2**(1): p. 5.
12. Hoenig, H., J.D.H. Taylor, and F.A. Sloan, *Does assistive technology substitute for personal assistance among the disabled elderly?* American Journal of Public Health, 2003. **93**(2): p. 330-337.
13. Maciejasz, P., et al., *A survey on robotic devices for upper limb rehabilitation.* Journal of NeuroEngineering and Rehabilitation, 2014. **11**(3).
14. Chen, L.-K.P., et al., *An evaluation of reachers for use by older persons with disabilities.* Assistive Technology, 1998. **10**(2): p. 113-125.
15. Römer, G.R.B.E., H.J.A. Stuyt, and A. Peters, *Cost-Savings and Economic Benefits due to the Assistive Robotic Manipulator (ARM).* Proceedings of the 2005 IEEE 9th International Conference on Rehabilitation Robotics, 2005.
16. Frappier, J., *Clinico-economic study of the JACO robotic arm for powered wheelchair users with upper-extremity disabilities.* by Data4Actions Inc. for Kinova, Canada, 2011.
17. Sugar, T.G., et al., *Design and Control of RUPERT: A Device for robotic upper extremity repetitive therapy.* IEEE TRANSACTIONS ON NEURAL SYSTEMS AND REHABILITATION ENGINEERING, 2007. **15**(3): p. 336-346.
18. Moromugi, S., et al., *Exotendon Glove System for Finger Rehabilitation after Stroke.* 2014. **7**: p. 93-102.
19. Lu, E.C., et al., *The development of an upper limb stroke rehabilitation robot: identification of clinical practices and design requirements through a survey of therapists.* Disabil Rehabil Assist Technol, 2011. **6**(5): p. 420-31.
20. Timmermans, A.A., et al., *Technology-assisted training of arm-hand skills in stroke: concepts on reacquisition of motor control and therapist guidelines for rehabilitation technology design.* J Neuroeng Rehabil, 2009. **6**: p. 1.
21. Schaechter, J.D., *Motor rehabilitation and brain plasticity after hemiparetic stroke.* Progress in Neurobiology, 2004. **73**(1): p. 61-72.
22. Ball, S.J., I.E. Brown, and S.H. Scott, *A planar 3DOF robotic exoskeleton for rehabilitation and assessment.* 2007: p. 4024-4027.
23. Prange, G.B., et al., *Preliminary findings of feasibility and compliance of technology-supported distal arm training at home after stroke.* Biosystems & Biorobotics 2014. **7**: p. 665-673

24. Kwakkel, G., B.J. Kollen, and H.I. Krebs, *Effects of Robot-Assisted Therapy on Upper Limb Recovery After Stroke: A Systematic Review.* Neurorehabilitation and Neural Repair, 2007. **22**(2): p. 111-121.
25. Prange, G.B., et al., *Systematic review of the effect of robot-aided therapy on recovery of the hemiparetic arm after stroke.* The Journal of Rehabilitation Research and Development, 2006. **43**(2): p. 171.
26. Balasubramanian, S., J. Klein, and E. Burdet, *Robot-assisted rehabilitation of hand function.* Current Opinion in Neurology, 2010. **23**(6): p. 661-670.
27. Radder B., P.-L.G.B., Kottink A.I.R., Gaasbeek L., Holmberg J., Meyer T., Buurke J.H., Rietman J.S. , *Preliminary Findings of Feasibility of a Wearable Soft-robotic Glove Supporting Impaired Hand Function in Daily Life - A Soft-robotic Glove Supporting ADL of Elderly People.* Proceedings of the International Conference on Information and Communication Technologies for Ageing Well and e-Health, 2016.
28. Radder, B., Kottink, A.I.R., van der Vaart, N., Oosting, D., Buurke, J.H., Nijenhuis, S.M., ... & Rietman, J.S., *User-centred input for a wearable soft-robotic glove supporting hand function in daily life.* 2015 IEEE International Conference on Rehabilitation Robotics (ICORR), 2015: p. 502-507.
29. Nilsson, M., *A Helping Hand: On Innovations for Rehabilitation and Assistive Technology.* 2013.
30. Hackel, M.E., Wolfe, G.A., Bang, S.M., & Canfield, J.S. , *Changes in hand function in the aging adult as determined by the Jebsen Test of Hand Function.* Physical Therapy, 1992. **72**(5): p. 373-377.
31. Jebsen, R.H., et al., *An objective and standardized test of hand function.* Archives of physical medicine and rehabilitation, 1969. **50**(6): p. 311.
32. Bohannon, R.W., *Hand-Grip Dynamometry Predicts Future Outcomes in Aging Adults.* Journal of geriatric physical therapy, 2008. **31**(1): p. 3-10.
33. Rijk, J.M., et al., *Prognostic value of handgrip strength in people aged 60 years and older: A systematic review and meta-analysis.* Geriatrics & Gerontology International, 2015.
34. Nicolay, C.W. and A.L. Walker, *Grip strength and endurance: Influences of anthropometric variation, hand dominance, and gender.* International Journal of Industrial Ergonomics, 2005. **35**(7): p. 605-618.
35. Desrosiers, J., et al., *Validation of the Box and Block Test as a measure of dexterity of elderly people: reliability, validity, and norms studies.* Archives of physical medicine and rehabilitation, 1994. **75**(7): p. 751-755.
36. Mathiowetz, V., Volland, G., Kashman, N., & Weber, K. , *Adult norms for the Box and Block Test of manual dexterity.* Am J Occup Ther, 1985. **39**(6): p. 386-391.
37. van der Lee, J.H., et al., *Clinimetric Properties of the Motor Activity Log for the Assessment of Arm Use in Hemiparetic Patients.* Stroke, 2004. **35**(6): p. 1410-1414.
38. Taub, E., et al., *Motor Activity Log (MAL) Manual.* 2011.
39. Aaronson, N.K., Muller, M., Cohen, P. D., Essink-Bot, M. L., Fekkes, M., Sanderman, R., ... & Verrips, E. , *Translation, validation, and norming of the Dutch language version of the SF-36 Health Survey in community and chronic disease populations.* Journal of clinical epidemiology, 1998. **51**(11): p. 1055-1068.
40. Brazier, J., Harper, R., Jones, N. M., O'cathain, A., Thomas, K. J., Usherwood, T., & Westlake, L., *Validating the SF-36 health survey questionnaire: new outcome measure for primary care.* BMJ: British Medical Journal, 1992. **305**(6846): p. 160-164.
41. Brooke, J., *SUS-A quick and dirty usability scale.* Usability evaluation in industry, 1996. **189**(194).
42. Markland, D. and L. Hardy, *On the factorial and construct validity of the Intrinsic Motivation Inventory: Conceptual and operational concerns.* Research Quarterly for exercise and Sport, 1997. **68**(1): p. 20-32.
43. McAuley, E., T. Duncan, and V.V. Tammen, *Psychometric properties of the Intrinsic Motivation Inventory in a competitive sport setting: A confirmatory factor analysis.* Research quarterly for exercise and sport, 1989. **60**(1): p. 48-58.
44. McAuley, E. and V.V. Tammen, *The effects of subjective and objective competitive outcomes on intrinsic motivation.* Journal of Sport and Exercise Psychology, 1989. **11**(1): p. 84-93.

45. Bower, C., H. Taheri, and E. Wolbrecht, *Adaptive control with state-dependent modeling of patient impairment for robotic movement therapy.* IEEE Int Conf Rehabil Robot, 2013. **2013**: p. 6650460.

15. Appendix 1

15.1 Subject characteristics part 1

Date (of inclusion)		.... - .... - ....
Name		
Address	Street + nr.	
	Postal code	
	City	
Telephone number		Home:<br><br>Mobile:
E-mail address		
Subject code		
Bank account number		
Km travel per visit		
Notes		

15.2 Subject characteristics part 2

Subject code	
Sex	Male / Female
Date of birth	.... - .... - ....
Age at inclusion	.... years
Impairment/Pathology	
Time since impairment	.... months
Most-affected body side	left / right
Dominant side before impairment	left / right
Weight	.... kg
Height	.... cm
Notes	

16. Appendix 2

16.1 Jebsen-Taylor Hand Function test

Materials: the JTHFT-kit, stopwatch, table and chair (with backside, preferably without armrests).

In the iH project, the JTHFT test will measure a) fine motor skills; (b) weighted functional tasks; and (c) non-weighted functional tasks [45]. The JTHFT consist of the following 7 tasks:

- Writing a short sentence (24 letters, 3rd grade reading difficulty)
- Turning over a 3x5 inch card
- Picking up small common objects (e.g., paper clips, coins and bottle caps)
- Simulated feeding (e.g. teaspoon with beans)
- Stacking checkers (test of eye-hand coordination)
- Picking up large light cans (e.g. empty cans)
- Picking up large heavy cans (450 g)

The JTHFT tasks are performed according to the instructions of Jebsen et al. 1969 [45].
The subject sits close to the table and the trunk must remain in contact with the back of the chair throughout testing. The distance between the subject and the activity should be a comfortable distance.

The time of each different task will be recorded in seconds and all times summed is the total score for the test. The participant will start after the researcher count up to 3 and in the meantime the researcher starts the stopwatch. The researcher stops the stopwatch when the subject finished the activity.

Number	Test	Time (seconds) without glove	Time (seconds) with glove
1	Writing a short sentence		
2	Turning over a 3x5 inch card		
3	Picking up small common objects		
4	Simulated feeding		
5	Stacking checkers		
6	Picking up large light cans		
7	Picking up large heavy cans		
	**Total score**		

17. Appendix 3

17.1 Box and Blocks Test

Benodigdheden: Box and Blocks kit met 150 blokken.

Voordat de test begint mag de deelnemer 15 seconden oefenen. Na deze oefenperiode kan de deelnemer nog instructies krijgen voordat de test begint. Voordat de test begint, zal de deelnemer zijn handen plaatsen naast de bak met blokken. Tijdens de test moet hij/zij zoveel mogelijk blokken in 1 minuut plaatsen in de lege bak er naast. De blokken mogen alleen 1 voor 1 verplaatst worden. De onderzoeker zal na de test het aantal blokken tellen dat verplaatst is. Als er meerdere blokken tegelijk verplaatst zijn moeten die van het totaal afgetrokken worden [36].

Subject code : ____
Dominante hand: Rechts / Links

Zonder iH AS: __________ Met iH AS: _______

18. Appendix 4

18.1 Motor Activity Log (MAL

De Motor Activity Log is een semi-gestructureerd interview die meet hoe vaak en hoe goed de patiënt de aangedane arm en hand gebruikt in het dagelijks leven. De patiënt wordt gevraagd hoe vaak hij/zij de aangedane arm gebruikt (AOU) en wat de kwaliteit van de beweging (QOM) is gedurende het uitvoeren van die dagelijkse activiteiten, waargenomen door de patiënt zelf.
De schalen zijn op aparte formulieren uitgeprint in liggen recht voor de patiënt tijdens het afnemen van de test.
De onderzoeker moet de patiënt helpen herinneren, dat het gaat om wat de patiënt daadwerkelijk in het dagelijks leven doet, niet wat hij denkt dat hij kan.
Begin met de AOU-schaal. De onderzoeker moet geen vraag stellen over de QOM als die bepaalde activiteit bij de AOU-schaal met 0 is beantwoord.
Voordat de test begint moet goed worden uitgelegd wat het verschil is tussen de AOU en QOM schaal, bijvoorbeeld:
U gebruikt uw aangedane arm zelden bij het pakken van een glas om iets te drinken, dus score 2. Maar, als u dit doet, dan doet u dat redelijk goed, dus een score 3.
Begrijpt u het verschil tussen de twee scores?
Zijn er verder nog vragen? Zo nee, dan gaan we nu beginnen met de vragenlijst.

Stap 1
De onderzoeker informeert of de activiteiten afgelopen week zijn uitgevoerd, met behulp van de volgende vraag:
'Als u kijkt naar de activiteiten die u de afgelopen week heeft uitgevoerd, heeft u toen uw aangedane arm en hand gebruikt bij…. **(noem de activiteit)*?'***
Als het antwoord nee is, vraag waarom de arm niet is gebruikt. Gebruik daarvoor de apart uitgeprinte lijst 1: mogelijke redenen waarom de aangedane arm niet is gebruikt tijdens een activiteit. Indien een patiënt de aangedane arm niet gebruikte voor een activiteit omdat het onmogelijk was (bv. Haren kammen voor een persoon die kaal is), geef dan de score NVT en laat deze vraag uit de test. Anders geef een score 0.

Stap 2
Het scoren van de Amount of Use – schaal. Stel de volgende vraag aan de patiënt:
'Hoe vaak doet de aangedane arm mee bij… **(noem de activiteit)*?'***
Geef een antwoord wat het beste bij u past. Gebruik daarbij de volgende schaal:
0. Aangedane arm doet **nooit** mee bij het uitvoeren van deze activiteit
1. Aangedane arm doet **zelden** mee bij het uitvoeren van deze activiteit
2. Aangedane arm doet **soms** mee bij het uitvoeren van deze activiteit
3. Aangedane arm doet **vaak** mee bij het uitvoeren van deze activiteit
   (ongeveer de helft als wat ik deed vóór de beroerte)
4. Aangedane arm doet **meestal** mee bij het uitvoeren van deze activiteit
   (ongeveer 3/4 van wat ik deed vóór de beroerte)
5. Aangedane arm doet **altijd** mee bij het uitvoeren van deze activiteit
   (hetzelfde als wat ik deed vóór de beroerte)

Stap 3

Het scoren van de Quality of Movement – schaal. Stel de volgende vraag aan de patiënt:

'Hoe goed* doet de aangedane arm mee bij…** **(noem de activiteit)*?'

Geef een antwoord wat het beste bij u past. Gebruik daarbij de volgende schaal:

0. Aangedane arm doet niet mee bij het uitvoeren van deze activiteit (**niet gebruikt**)
1. Aangedane arm doet heel iets mee bij het uitvoeren van deze activiteit, maar was niet noemenswaardig (**heel slecht**)
2. Aangedane arm doet iets mee bij het uitvoeren van deze activiteit, maar had hulp nodig van de niet-aangedane arm of bewoog heel langzaam of met moeite (**slecht**)
3. Aangedane arm doet mee bij het uitvoeren van deze activiteit, maar bewegingen zijn langzaam of zijn gemaakt met weinig resultaat (**redelijk**)
4. Aangedane arm doet goed mee bij het uitvoeren van deze activiteit, maar de bewegingen zijn nog niet zo snel of netjes als normaal (**bijna normaal**)
5. Aangedane arm doet net zo goed mee bij het uitvoeren van deze activiteit als voor de beroerte (**normaal**)

Indien de onderzoeker niet zeker is over een antwoord dat gegeven is door de patiënt, vraag de patiënt dan om de activiteit voor the doen, door te zeggen:

'Kunt u misschien laten zien hoe u die activiteit zou doen?'

Of ga verder in discussie over die activiteit:

'U hebt op die activiteit een 3 (redelijk) gescoord voor hoe goed u de arm gebruikt. Echter, u beweegt uw arm erg langzaam tijdens het uitvoeren van de activiteit, dus zou het misschien meer een score 2 zijn: slecht. Vindt u niet?'

Bij de evalutie metingen post-training (ET1) is het handig om de score van de vorige meting erbij te hebben als referentie. Dit om aan te geven dat er eventueel een verandering is. Maar zorg dat de patiënt deze lijst niet ziet! Als een score verandert ten opzichte van de vorige keer (hoger of lager), stel de volgende vragen:

'Vandaag hebt u bij deze activiteit hoger/lager geantwoord ten opzichte van de vorige keer. Waarom is dat denkt u? Heeft er een echte verandering plaatsgevonden ten opzichte van de vorige keer?'

18.1.1 Score formulier

**Aangedane arm gebruikt bij deze activiteit?**	**Activiteit**	**Score A (AOU)**	**Score B (QOM)**
JA / NEE	01. Zich vasthouden bij staan		
JA / NEE	02. Arm in de mouw doen van een kledingstuk		
JA / NEE	03. Iets in de hand van de ene naar de andere plaats dragen		
JA / NEE	04. Eten met mes en vork		
JA / NEE	05. Haar kammen		
JA / NEE	06. Kopje aan het oor oppakken		
JA / NEE	07. Handenarbeid/kaartspelen/hobby's		
JA / NEE	08. Vasthouden/bladzijden omslaan van een boek/tijdschrift/krant		
JA / NEE	09. Handdoek gebruiken om gezicht/ander lichaamsdeel af te drogen		
JA / NEE	10. Glas optillen		
JA / NEE	11. Tandenpoetsen		
JA / NEE	12. Scheren/make-up		
JA / NEE	13. Deur openen met sleutel		
JA / NEE	14. Brief schrijven/typen		
JA / NEE	15. Koffie/thee inschenken		
JA / NEE	16. Fruit schillen		
JA / NEE	17. Een nummer intoetsen bij telefoneren		
JA / NEE	18. Een raam openen/sluiten		
JA / NEE	19. Een brief openen		
JA / NEE	20. Geld uit portemonnee pakken		
JA / NEE	21. Knopen van kleding losmaken		
JA / NEE	22. Knopen van kleding sluiten		
JA / NEE	23. Rits openmaken		
JA / NEE	24. Rits sluiten		
JA / NEE	25. Nagels knippen		
JA / NEE	26. Andere activiteiten, n.l		
Aantal JA:	**Totaal score** (= som alle activiteiten)		
	**Sum score** (= totaal score / aantal activiteiten JA)		

18.1.2 Extra aantekeningen voor bij het scoren

Na het afnemen van de MAL, een gemiddelde MAL score wordt berekend zowel voor de AOU en QOM schaal. Als een patiënt een vraag beantwoord heeft met NEE (ik heb de taak niet gedaan), probeer dan te achterhalen waarom niet. Als het voor de patiënt onmogelijk is om de activiteit uit te voeren (bv. Haren kammen bij een kale man), noteer NVT en zal de vraag vervallen. Bereken de gemiddelde score dan zonder die vraag (bv. Totaal score delen door 25 in plaats van 26). Noteer alleen een score NVT als u zeker weet dat de activiteit onmogelijk is om uit te voeren. Noteer anders een score 0 bij een vraag die met nee is beantwoord (dan wordt de gemiddelde score gewoon berekend door het te delen door 26).
Als een patiënt een bepaalde activiteit niet in staat was om te doen, maar de vorige keer nog wel, neem dan de score over van de vorige evaluatie meting. Bijvoorbeeld, een patiënt was in staat om een koelkast open te maken tijdens de eerste evaluatie meting (T1), maar gedurende een tijd heeft de patiënt op een hotelkamer gezeten waarbij hij geen beschikking had over een koelkast.
Als een patiënt aangeeft geen enkele activiteit te kunnen met de aangedane arm en hand, accepteer dit dan niet zo maar. Begin met de test en neem de eerste 10 vragen af. Indien alle activiteiten met 0 worden beantwoord, dan kunt u aannemen dat de rest van de test ook met 0 zal worden beantwoord, en kunt u stoppen met de test.

Score berekening:

- Scores worden apart berekend voor AOU en QOM.
- Tel de test scores van alle activiteiten bij elkaar op, apart voor AOU en QOM (kolom A en B). Dit zijn de totaalscores.
- Om de som score per schaal te berekenen, wordt de som van alle activiteiten gedeeld door het aantal activiteiten dat werkelijk is uitgevoerd (het aantal activiteiten dat met JA beantwoordt is). Het eindresultaat is twee gemiddelde scores, een voor AOU en een voor QOM.

Interpretatie:

In de literatuur zijn geen minimal clinically important differences (MCID) gevonden voor de MAL. Op basis van klinische ervaring en schattingen voor gelijkwaardige uitkomstmaten, zal de MCID worden gezet op 10% van de totale range van de schaal. Dat wil zeggen, een MCID van 0.50 punten (van der Lee, 1999).

19. Appendix 5

19.1 SF-36

In deze vragenlijst wordt naar uw gezondheid gevraagd. Wilt u elke vraag beantwoorden door het juiste hokje aan te kruisen. Wanneer u twijfelt over het antwoord op een vraag, probeer dan het antwoord te geven dat het meest van toepassing is

1. Wat vindt u, over het algemeen genomen, van uw gezondheid?

uitstekend ☐
zeer goed ☐
goed ☐
matig ☐
slecht ☐

2. *In vergelijking met een jaar geleden*, hoe zou u *nu* uw gezondheid in het algemeen beoordelen?

veel beter dan een jaar geleden ☐
iets beter dan een jaar geleden ☐
ongeveer hetzelfde als een jaar geleden ☐
iets slechter dan een jaar geleden ☐
veel slechter dan een jaar geleden ☐

3. De volgende vragen gaan over dagelijkse bezigheden. Wordt u door uw gezondheid *op dit moment* beperkt bij deze bezigheden? Zo ja, in welke mate?

	**Ja, ernstig beperkt**	**Ja, een beetje beperkt**	**Nee, helemaal niet beperkt**
Forse inspanning<br>a. zoals hardlopen, zware voorwerpen tillen, inspannend sporten	☐	☐	☐
Matige inspanning<br>b. zoals het verplaatsen van een tafel, stofzuigen, fietsen	☐	☐	☐
Tillen of boodschappen dragen	☐	☐	☐
Een paar trappen lopen	☐	☐	☐
Eén trap oplopen	☐	☐	☐
Buigen, knielen of bukken	☐	☐	☐
Meer dan een kilometer lopen	☐	☐	☐
Een halve kilometer lopen	☐	☐	☐
Honderd meter lopen	☐	☐	☐
Uzelf wassen of aankleden	☐	☐	☐

4. Had u, ten gevolge van uw lichamelijke gezondheid, *de afgelopen 4 weken* één van de volgende problemen bij uw werk of andere dagelijkse bezigheden?

	Ja	Nee
U heeft *minder tijd* kunnen besteden aan werk of andere bezigheden	☐	☐
U heeft *minder bereikt* dan u zou willen	☐	☐
U was beperkt in het *soort* werk of het soort bezigheden	☐	☐
U had moeite met het werk of andere bezigheden (het kostte u bijvoorbeeld extra inspanning)	☐	☐

5. Had u, ten gevolge van een emotioneel probleem (bijvoorbeeld doordat u zich depressief of angstig voelde), *de afgelopen 4 weken* één van de volgende problemen bij uw werk of andere dagelijkse bezigheden?

	Ja	Nee
U heeft *minder tijd* kunnen besteden aan werk of andere bezigheden	☐	☐
U heeft *minder bereikt* dan u zou willen	☐	☐
U heeft het werk of andere bezigheden niet zo zorgvuldig gedaan als u gewend bent	☐	☐

6. In hoeverre heeft uw lichamelijke gezondheid of hebben uw emotionele problemen u *de afgelopen 4 weken* belemmerd in uw normale sociale bezigheden met gezin, vrienden, buren of anderen?

Helemaal niet ☐
Enigszins ☐
Nogal ☐
Veel ☐
Heel erg veel ☐

7. Hoeveel lichamelijke pijn had u *de afgelopen vier weken*?

Geen ☐
Heel licht ☐
Licht ☐
Nogal ☐
Ernstig ☐
Heel ernstig ☐

8. In welke mate heeft pijn u *de afgelopen vier weken* belemmerd bij uw normale werkzaamheden (zowel werk buitenshuis als huishoudelijk werk)?

Helemaal niet ☐
Een klein beetje ☐
Nogal ☐
Veel ☐
Heel erg veel ☐

9. Deze vragen gaan over hoe u zich *de afgelopen 4 weken* heeft gevoeld. Wilt u bij elke vraag het antwoord aankruisen dat het beste aansluit bij hoe u zich heeft gevoeld.
Hoe vaak gedurende *de afgelopen 4 weken*:

	Voortdurend	Meestal	Vaak	Soms	Zelden	Nooit
voelde u zich levenslustig?	☐	☐	☐	☐	☐	☐
voelde u zich erg zenuwachtig?	☐	☐	☐	☐	☐	☐
zat u zo erg in de put dat niets u kon opvrolijken?	☐	☐	☐	☐	☐	☐
voelde u zich kalm en rustig?	☐	☐	☐	☐	☐	☐
voelde u zich erg energiek?	☐	☐	☐	☐	☐	☐
voelde u zich neerslachtig en somber?	☐	☐	☐	☐	☐	☐
voelde u zich uitgeblust?	☐	☐	☐	☐	☐	☐
voelde u zich gelukkig?	☐	☐	☐	☐	☐	☐
voelde u zich moe?	☐	☐	☐	☐	☐	☐

10. *Hoe vaak* hebben uw lichamelijke gezondheid of emotionele problemen gedurende *de afgelopen 4 weken* uw sociale activiteiten (zoals bezoek aan vrienden of naaste familieleden) belemmerd?

Voortdurend ☐
Meestal ☐
Soms ☐
Zelden ☐
Nooit ☐

11. Wilt u het antwoord kiezen dat het beste weergeeft hoe juist of onjuist u elk van de volgende uitspraken voor uzelf vindt.

	Volkomen juist	Grotendeels juist	Weet ik niet	Grotendeels onjuist	Volkomen onjuist
Ik lijk gemakkelijker ziek te worden dan andere mensen	☐	☐	☐	☐	☐
Ik ben net zo gezond als andere mensen die ik ken	☐	☐	☐	☐	☐
Ik verwacht dat mijn gezondheid achteruit zal gaan	☐	☐	☐	☐	☐
Mijn gezondheid is uitstekend	☐	☐	☐	☐	☐

20. Appendix 6

20.1 System Usability Scale

Instructie: Geef voor iedere stelling aan in welke mate u met deze stelling oneens / eens bent door het hokje aan te kruizen wat het beste past bij uw mening over het iH systeem. Probeer op elke vraag meteen een antwoord te geven in plaats van lang na te denken over de vraag.

		**In sterke mate mee oneens**				**In sterke mate mee eens**
**1**	Ik denk dat ik het iH systeem vaker wil gebruiken	☐	☐	☐	☐	☐
**2**	Ik vind het iH systeem onnodig ingewikkeld	☐	☐	☐	☐	☐
**3**	Ik vind het iH systeem gemakkelijk te gebruiken	☐	☐	☐	☐	☐
**4**	Ik denk dat ik hulp nodig heb van een technisch persoon om het iH systeem te kunnen gebruiken	☐	☐	☐	☐	☐
**5**	Ik vind de verschillende functionaliteiten van het iH systeem goed geïntegreerd.	☐	☐	☐	☐	☐
**6**	Ik denk dat er te veel tegenstrijdigheden in het iH systeem zitten	☐	☐	☐	☐	☐
**7**	Ik kan me voorstellen dat de meeste personen snel leren hoe ze het iH systeem moeten gebruiken	☐	☐	☐	☐	☐
**8**	Ik vind het iH systeem erg omslachtig / lastig in gebruik	☐	☐	☐	☐	☐
**9**	Ik voelde me erg zelfverzekerd tijdens het gebruiken van het iH systeem	☐	☐	☐	☐	☐
**10**	Ik moet nog veel leren voordat ik het iH systeem kan gebruiken	☐	☐	☐	☐	☐

Ruimte voor eventuele opmerkingen:

Deze vragenlijst in gebasseerd op de System Usability Scale(SUS), ontwikkeld door John Brooke (1995). © Digital Equipment Corporation, 1986.

21. Appendix 7

21.1 Intrinsic Motivation Inventory

Hieronder vindt u een lijst met 34 vragen welke betrekking hebben op uw ervaringen met het iH systeem.

Voor ieder van de volgende stellingen kunt u aangeven in hoeverre dit voor u geldt, door het vakje aan te kruisen wat het beste klopt met wat u vindt of voelt, volgens onderstaande uitleg:

1	2	3	4	5	6	7
Helemaal niet mee eens			*Een beetje mee eens*			*Helemaal mee eens*

Nr. VL	*A*	**Interesse/plezier**	1	2	3	4	5	6	7
28	*1*	Het gebruiken van het iH systeem boeide mij totaal niet.							
10	*2*	Ik vond het iH systeem erg interessant.							
26	*3*	Ik besefte me bij het gebruiken van het iH systeem hoeveel ik ervan kon genieten.							
1	*4*	Ik beleefde veel plezier aan het gebruiken van het iH systeem.							
19	*5*	Ik vond het saai om het iH systeem te gebruiken.							
17	*6*	Het gebruiken van het iH systeem gaf me voldoening.							
30	*7*	Het gebruiken van het iH systeem was erg leuk om te doen.							

Nr. VL	*B*	**Waargenomen competentie**	1	2	3	4	5	6	7
18	*1*	Nadat ik het iH systeem een tijdje gebruikt had, had ik het gevoel dat ik het best goed kon gebruiken.							
27	*2*	Ik vond mezelf niet zo goed in het gebruiken van het iH systeem.							
2	*3*	Ik denk dat ik het iH systeem beter kon gebruiken dan andere deelnemers aan het onderzoek (mensen van mijn leeftijd met een beroerte).							
11	*4*	Ik denk dat ik vrij goed was in het gebruiken van het iH systeem.							
34	*5*	Ik ben over het algemeen tevreden over mijn prestaties met het gebruiken van het iH systeem.							
29	*6*	Ik vond mezelf vrij vaardig bij het gebruiken van het iH systeem.							

Nr. VL	C	Inzet/belangrijkheid	1	2	3	4	5	6	7
20	*1*	Ik vond het belangrijk om goed mijn best te doen bij het gebruiken van het iH systeem.							
25	*2*	Ik heb niet erg mijn best gedaan om het iH systeem goed te gebruiken.							
3	*3*	Ik heb niet veel energie in het gebruiken van het iH systeem gestoken.							
31	*4*	Ik heb met veel inzet het iH systeem gebruikt.							
9	*5*	Ik deed erg mijn best bij het gebruiken van het iH systeem.							

Nr. VL	D	Waargenomen keuze	1	2	3	4	5	6	7
14	*1*	Ik had eigenlijk geen keus bij het gebruiken van het iH systeem.							
21	*2*	Ik heb het idee dat ik zelf een keus had bij het gebruiken van het iH systeem.							
8	*3*	Ik gebruikte het iH systeem omdat ik wel moest.							
12	*4*	Ik kreeg het gevoel dat ik verplicht het iH systeem moest gebruiken.							
4	*5*	Ik gebruikte het iH systeem omdat ik geen keus had.							
16	*6*	Ik heb het gevoel dat ik geen eigen keuze had bij het gebruiken van het iH systeem.							
24	*7*	Ik gebruikte het iH systeem omdat ik dat wilde doen.							

Nr. VL	E	Druk/spanning	1	2	3	4	5	6	7
5	*1*	Ik was erg ontspannen tijdens het gebruiken van het iH systeem.							
13	*2*	Ik voelde erg veel spanning bij het gebruiken van het iH systeem.							
32	*3*	Ik voelde druk tijdens het gebruiken van het iH systeem.							
7	*4*	Ik voelde me helemaal niet nerveus terwijl ik het iH systeem gebruikte.							
23	*5*	Ik was gespannen bij het gebruiken van het iH systeem.							

Nr. VL	F	Waarde/nut	1	2	3	4	5	6	7
22	*1*	Het gebruiken van het iH systeem was waardevol voor mij, daarom zou ik het graag nog eens gebruiken.							
33	*2*	Ik vind het gebruiken van het iH systeem belangrijk.							
15	*3*	Ik geloof dat het gebruiken van het iH systeem voordelen oplevert voor mij.							
6	*4*	Ik geloof dat het gebruiken van het iH systeem waardevol voor mij kan zijn.							

22. Appendix 8

22.1 Semi-structured interview about user experience – Assistive group

We strive to develop products that meet your needs. Therefore we highly appreciate that you agreed to participate in the testing of the ironHand system. With the following questions we would like to receive your feedback about the usage and effect of the ironHand system that you tested during the last weeks.
Your participation helps us to continuously improve our products within the clinical environment.
Thank you very much for your feedback!

**Subject code**	

1) **What do you think about the ironHand system and the support it delivered (grade 0-10)?**
   Grade:
   ……………………………………………………………………………………………
   ………

2) **How was it to put on/take off the ironHand system?**
   - Was it easy or hard?
     ....
     ....
     ....
   - Did you manage it by yourself?
     ....
     ....
     ....
   - What was it that made it difficult to put the glove on? Suggestions for improvement?
     ....
     ....
     ....
     ......

3) **What do you think about the ease of use of the system?**

....
....
....
....
....
....

4) **Did you have to involve your carer (if you have one) when using the ironHand system? Did they assist you in any way? If so/not, could you please explain why?**

....
....
....
....
....
....

5) **Did the ironHand system fit into your physical, personal and social environment?**

....
....
....
....
....
....

<u>If the participant is still working (including voluntary work), please also fill in questions 6-8! If not, go to question 9.</u>

6) **Have you used the ironHand system at work in the last four weeks?**

Yes / No

Please explain:………………………………………………………………………………..
...
………………………………………………………………………………………
……………

7) **Did the ironHand system fit into your working environment?**

Yes / No

Positive
aspects…………………………………………………………………………….....
…………………………………………………………………………………………
…………………………………………………………………………………………
…………………………
Negative aspects:
………………………………………………………………………………
…………………………………………………………………………………………
…………………………………………………………………………………………
…………………………

8) **If the system was used during work, for how many days/hours and during which work-related activities was the ironHand system used?**
Number of days/hours the ironHand system was used last 4 weeks in work related activities
(estimation):…………………………………………………………………….................
....

Work-related
activities:…………………………………………………………………………
…………………………………………………………………………………………
…………………………………………………………………………………………
…………………………

9) **Overall, what were the best things about the prototype?**
…………………………………………………………………………………………
…………………………………………………………………………………………
…………………………………………………………………………………………
…………………………………………………………………………………………
…………………………………………………………………………………………
……………………………………………………………………

10) **Overall, what were the worst things about the prototype? So what needs most improvements?**
…………………………………………………………………………………………
…………………………………………………………………………………………
…………………………………………………………………………………………

……………………………………………………………………………………………

……………………………………………………………………………………………

……………………………………………

11) If this was possible, would you buy an ironHand system?

Yes / No

- If yes, what would be the maximum price you want to pay for the ironHand system:

  ☐ 500 Euro

  ☐ 1000 Euro

  ☐ 1500 Euro

  ☐ Other,

……………………………………………………………………………………………

- If no, what is the main reason that you didn’t want to buy the ironHand system?

……………………………………………………………………………………………

……………………………………………………………………………………………

……………………………………………………………………………………………

……………………………………………

Thank you very much for your participation!

22.2 Semi-structured interview about user experience – therapeutic group

We strive to develop products that meet your needs. Therefore we highly appreciate that you agreed to participate in the testing of the ironHand system. With the following questions we would like to receive your feedback about the usage and effect of the ironHand system that you tested during the last weeks.
Your participation helps us to continuously improve our products within the clinical environment.
Thank you very much for your feedback!

**Subject code**	

12) What do you think about the ironHand therapeutic system (grade 0-10)?

Grade:
…………………………………………………………………………………………
…

13) How was it to put on/take off the glove of the ironHand therapeutic system?

- Was it easy or hard?
  ....
  ....
  ....
- Did you manage it by yourself?
  ....
  ....
  ....
- What was it that made it difficult to put the glove on? Suggestions for improvement?
  ....
  ....
  ....
  ......

14) What do you think about the ease of use of the ironHand therapeutic system?

....

....

....

....

....

....

15) **Did you have to involve your carer (if you have one) when using the ironHand system? Did they assist you in any way? If so/not, could you please explain why?**

....

....

....

....

....

....

16) **Did the ironHand therapeutic system fit into your physical, personal and social environment?**

....

....

....

....

....

....

17) **If this was possible, would you want to continue your therapy at home with the ironHand therapeutic system?**

Yes / No

Please explain:...

....

....

....

......

<u>Therapeutic exercises</u>

1. **My favorite exercise was:**

   ☐ Submarine

   ☐ High flyer

   ☐ Birds on strings

2. **The most important aspect of the exercise was:**

   ☐ the exercises stimulated me to do specific arm/hand exercises

   ☐ the exercises motivated me to play the games in itself

   ☐ the exercises didn’t facilitate using my arm/hands at all

3. **The exercises helped me to alleviate my condition**

   0 ☹ ——————————————— 10 ☺

4. **I liked the therapeutic goal of the exercises**

   0 ☹ ——————————————— 10 ☺

5. **I understood the exercises**

   0 ☹ ——————————————— 10 ☺

6. **I was motivated through the exercises**

   0 ☹ ——————————————— 10 ☺

7. **I think the exercises were adapted to my abilities**

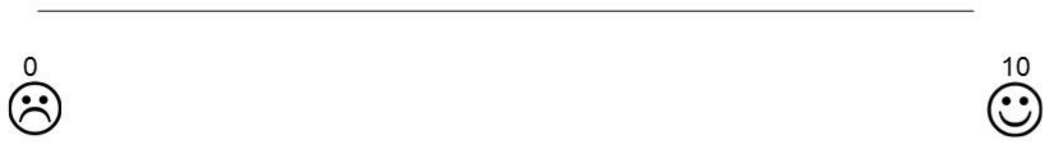

8. **Overall, I am satisfied with the exercises provided in the ironHand therapeutic system**

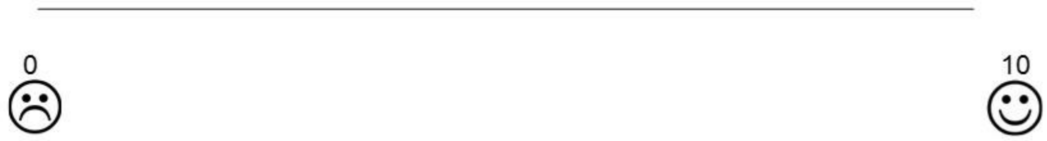

9. **What kind of enhancements to the current exercises could you imagine?**

…………………………………………………………………………………………

…………………………………………………………………………………………

…………………………………………………………………………………………

………………………………………

10. **What kind of additional exercises could you imagine, that target your problems?**

…………………………………………………………………………………………

…………………………………………………………………………………………

…………………………………………………………………………………………

………………………………………

11. **Other thoughts that you would like to share?**

…………………………………………………………………………………………

…………………………………………………………………………………………

…………………………………………………………………………………………

………………………………………

<u>Graphical User Interface</u>

12. **I think the computer programme was easy to use:**

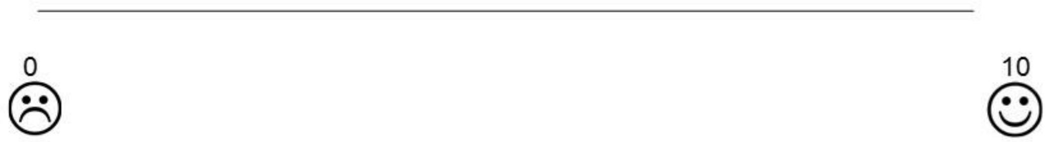

13. Do you prefer to have a results section within the ironHand therapeutic system in which you can see the (progression in) results of the specific hand exercises (e.g. score, number of hand movements etc.)?

Yes/No

Please explain:……………………………………………………………………………........

……

14. Do you prefer that your therapist is also informed about these results?

Yes/No

Please explain:……………………………………………………………………………........

……

15. Do you wish to receive feedback from the ironHand therapeutic system and/or from your therapist about the results?

☐ Only feedback from the ironHand therapeutic system

☐ Only feedback from your therapist

☐ Feedback from both

16. What kind of enhancements to how you use the programme could you imagine?

…………………………………………………………………………………………

…………………………………………………………………………………………

…………………………………………………………………………………………

………………………………………

17. Other thoughts you would like to share?

……………………………………………………………………………………………
……………………………………………………………………………………………
……………………………………………………………………………………………
………………………………………

18. If this was possible, would you buy an ironHand therapeutic system?

Yes / No

- If yes, what would be the maximum price you want to pay for the ironHand system:

  ☐ 500 Euro

  ☐ 1000 Euro

  ☐ 1500 Euro

  ☐ Other,

…………………………………………………………………………………..........

- If no, what is the main reason that you didn't want to buy the ironHand therapeutic system?
